# WOODIV, a database of occurrences, functional traits, and phylogenetic data for all Euro-Mediterranean trees

**DOI:** 10.1038/s41597-021-00873-3

**Published:** 2021-03-23

**Authors:** Anne-Christine Monnet, Kévin Cilleros, Frédéric Médail, Marwan Cheikh Albassatneh, Juan Arroyo, Gianluigi Bacchetta, Francesca Bagnoli, Zoltán Barina, Manuel Cartereau, Nicolas Casajus, Panayotis Dimopoulos, Gianniantonio Domina, Aggeliki Doxa, Marcial Escudero, Bruno Fady, Arndt Hampe, Vlado Matevski, Stephen Misfud, Toni Nikolic, Daniel Pavon, Anne Roig, Estefania Santos Barea, Ilaria Spanu, Arne Strid, Giovanni Giuseppe Vendramin, Agathe Leriche

**Affiliations:** 1 Aix Marseille Univ, Avignon Univ, CNRS, IRD, IMBE. Technopôle de l’Arbois-Méditerranée, cedex 4, BP 80, 13 545 Aix-en-Provence, France; 2 Institut de Systématique, Evolution, Biodiversité (ISYEB), Muséum national d’Histoire naturelle (MNHN), CNRS, Sorbonne Université, EPHE, Université des Antilles, Paris, France; 3 Department of Plant Biology and Ecology, University of Seville, Seville, Spain; 4 Department of Life and Environmental Sciences, University of Cagliari, Viale Sant’Ignazio da Laconi 13, Cagliari, Italy; 5 National Research Council, Institute of Biosciences and Bioresources, 50019 Sesto Fiorentino, (FI) Italy; 6 Department of Botany, Hungarian Natural History Museum, Pf. 137, Budapest, 1431 Hungary; 7 FRB-CESAB, 5 rue de l’Ecole de Médecine, 34000 Montpellier, France; 8 Department of Biology, Laboratory of Botany, University of Patras, 26504 Patras, Greece; 9 Department of Agriculture, Food and Forest Sciences, University of Palermo, Viale delle Scienze bldg. 4, 90128 Palermo, Italy; 10 Statistical Learning Lab, Institute of Applied and Computational Mathematics, Foundation for Research and Technology-Hellas (FORTH), Ν. Plastira 100, Vassilika Vouton, GR - 700 13 Heraklion, Crete Greece; 11 INRAE, UR629, Ecologie des forêts méditerranéennes, Avignon, France; 12 INRAE, Univ. Bordeaux, BIOGECO, F-33610 Cestas France; 13 Macedonian Academy of Sciences and Arts, Krste Misirkov 2, 1000 Skopje, Republic of Macedonia; 14 EcoGozo, Regional Development Directorate - Ministry for Gozo, Flat 6, Sunset Court B, Triq Marsalforn, Xaghra, Gozo Malta; 15 Department of Botany, Faculty of Science, University of Zagreb, Zagreb, Croatia; 16 Bakkevej 6, 5853 Ørbæk, Denmark

**Keywords:** Conservation biology, Community ecology, Biodiversity, Forest ecology, Biogeography

## Abstract

Trees play a key role in the structure and function of many ecosystems worldwide. In the Mediterranean Basin, forests cover approximately 22% of the total land area hosting a large number of endemics (46 species). Despite its particularities and vulnerability, the biodiversity of Mediterranean trees is not well known at the taxonomic, spatial, functional, and genetic levels required for conservation applications. The WOODIV database fills this gap by providing reliable occurrences, four functional traits (plant height, seed mass, wood density, and specific leaf area), and sequences from three DNA-regions (*rbc*L, *mat*K, and *trn*H-*psb*A), together with modelled occurrences and a phylogeny for all 210 Euro-Mediterranean tree species. We compiled, homogenized, and verified occurrence data from sparse datasets and collated them on an INSPIRE-compliant 10 × 10 km grid. We also gathered functional trait and genetic data, filling existing gaps where possible. The WOODIV database can benefit macroecological studies in the fields of conservation, biogeography, and community ecology.

## Abstract (summary)

**Table:** 

Measurement(s)	occurrence • Trait • DNA
Technology Type(s)	Sampling • digital curation • DNA sequencing assay
Sample Characteristic - Organism	Viridiplantae • trees
Sample Characteristic - Environment	forest biome
Sample Characteristic - Location	Mediterranean Region • Europe

Machine-accessible metadata file describing the reported data: 10.6084/m9.figshare.13553519

## Background & Summary

The forests of the Euro-Mediterranean Basin are home to 245 indigenous tree taxa (species and subspecies), 44 of which are cryptic (as defined by Médail *et al*.^1^ *i.e*. trees commonly considered as bushes with poorly defined multicolous stems but which can form true trees under certain environmental conditions), and a high number of endemics. The production of woody forest products represents 35% of the total economic value of Mediterranean forests^2^.

Although critical for biogeographical and conservation applications, data on the occurrence, functional traits, and phylogeny of the tree species of the Euro-Mediterranean region (as defined by Médail *et al*.^1^ from Portugal to Greece, including islands) are still sparse, not readily available, and present heterogeneous spatial and taxonomic coverage and resolution.

The current occurrence data available from various sources in different countries present challenges related to: (1) Taxonomy: not all sources use the same taxonomic reference to name the recorded species^3^ and present the same level of updating regarding recently described taxa Médail *et al*.^1^ (2) Spatial resolution: the spatial grain of the data varies (from georeferenced coordinates for single records to data gridded at varying resolutions); (3) Accessibility: the collection of species occurrence data is generally managed at the national or regional scale. Scientific and naturalist communities support and promote increasing data-sharing habits, and some data are publicly available through dedicated platforms. However, many local data remain inaccessible and/or non-digitalized. Gathering them requires identifying local datasets and/or contacting local flora specialists. Remaining regional or national gaps can be filled only by collecting data in the field or through expert knowledge; (4) Reliability: the reliability of the different sources is highly variable, especially information from biodiversity data portals^4^. For conservation and biogeographical purposes, it is crucial to detect and discard doubtful occurrences. The introduced or native status of a species is very informative but not always explicitly indicated.

Functional and genetic diversities are recognized as important components of biodiversity, implying that an effective conservation strategy should rely on the evolutionary processes in habitats, especially in the face of global change^5–8^.

Functional traits are important proxies to understand the relationships between biodiversity, ecosystem functioning, and environmental constraints. Over the last two decades, important efforts have been devoted to the centralization of plant traits in curated databases through international networks of plant scientists (*e.g*. TRY database^9^). However, these databases on functional traits are often biased toward guilds or species of specific interest (in trees, for instance, on economically important forestry species). Challenges 1, 3, and 4 identified above therefore also apply to tree functional trait data.

Measuring phylogenetic diversity is a potentially powerful way to explain the role of abiotic and biotic factors on community species and biogeographic histories in communities^10,11^. Many economically or ecologically important tree species of the Mediterranean Basin have undergone molecular genetic analyses and several sequences are often available for them^12^. For other species, sequence data are missing or sparse, requiring new field or herbarium sampling, DNA extraction and sequencing.

The WOODIV project funded by the FRB-CESAB and the LabEx OT-Med aimed to collate, homogenize, and verify datasets on tree species, their functional traits, and phylogeny, from existing but sparse datasets and complete them where possible (new data collected) for the Euro-Mediterranean area. The WOODIV database provides reliable occurrences, functional traits, and sequences for three DNA regions, together with the modelled occurrences and a phylogeny for the 210 Euro-Mediterranean tree species identified by Médail *et al*.^1^, including the 44 cryptic tree species which are often neglected in existing forest databases. The native or introduced status of a species in each location is also provided. This information combined with the number of considered taxa and sources of data, the inclusion of Mediterranean islands, and its resolution (10 × 10 km reference grid), result in a database of high significance and interest for macroecological studies in the Euro-Mediterranean area in the fields of conservation, biogeography, and community ecology.

## Methods

The geographic area covered by the WOODIV database is the Euro-Mediterranean region, as defined by Médail *et al*.^1^. The northern Mediterranean region was selected following the definition of terrestrial ecoregions of the world by Olson *et al*.^13^. The study area covers all or part of the following countries and islands: Albania, Croatia, Cyprus, France, Greece, Italy, Malta, Montenegro, Portugal, Slovenia, Southern Macedonia, and Spain, including the Balearic archipelago, Corsica, Sardinia, Sicily, and Crete.

We focused on the 245 tree taxa (210 species and 35 subspecies) identified in the Euro-Mediterranean checklist from Médail *et al*.^1^. These taxa belong to 33 families and 64 genera and include 46 endemics (as defined by Médail *et al*.^1^, *i.e*. range-restricted taxa in and outside of the study area).

### Observed occurrence data

We collected tree occurrence data (at the species or subspecies level) from 23 sources: national databases and floras, regional databases, and publications (Table 1). Some records still unpublished were specifically provided at the grid level for this project by experts for southern Macedonia, Malta, Montenegro, and Sicily (four sources, Table 1).

**Table 1:** Sources of the occurrence records, giving the name of the dataset (Source name; ined. if unpublished), the Type of data (records with geographic coordinates (records), records at the grid level (gridded records), or atlas-type (atlas) data), and the Countries/Islands covered by the source.

Ref	Source name	Type of data	Countries	Nb taxa	Nb rec	Nb cells	% taxa	% rec	% cells
1	Distribution atlas of vascular plants in Albania	atlas	Albania	103	5032	291	50.74	0.4	2.9
2	Flora Croatica Database	records	Croatia	117	41154	294	57.64	3.3	2.93
3	ined.	records	Cyprus	6	677	27	2.96	0.05	0.27
4	EUFGIS Genetic Conservation Units	records	Europe	23	195	148	11.33	0.02	1.47
5	GBIF	records	Cyprus	22	95	33	10.84	0.01	0.33
6	Conservatoire botanique national alpin (CBNA)	records	France	94	57369	163	46.31	4.59	1.62
7	Conservatoire botanique national de Corse (CBNC)	records	France, Corsica	80	24462	120	39.41	1.96	1.19
8	Conservatoire botanique national du Massif Central (CBNMC)	records	France	87	39612	53	42.86	3.17	0.53
9	IGN Inventaire Forestier	records	France, Corsica	102	68327	749	50.25	5.47	7.46
10	Conservatoire botanique national méditerranéen de Porquerolles (CBNMed)	records	France	111	345623	638	54.68	27.68	6.35
11	Flora Hellenica Database	atlas	Greece, Crete	139	34900	1618	68.47	2.79	16.11
12	GBIF	records	Portugal, Spain, Balearic Islands	106	554515	4993	52.22	44.41	49.72
13	Tentamen Florae Aeolicae	records	Eolian islands	46	246	21	22.66	0.02	0.21
14	CNR data	records	Italy	5	203	182	2.46	0.02	1.81
15	EU-Forest	atlas	Italy	79	12333	1362	38.92	0.99	13.56
16	INFC 2015	records	Italy	71	12332	1369	34.98	0.99	13.63
17	VegItaly	records	Italy	93	27421	681	45.81	2.2	6.78
18	WikiPlantBase # Liguria, # Toscana, # Sardegna, # Sicily	records	Italy	120	13303	506	59.11	1.07	5.04
19	ined.	gridded records	Macedonia	99	3461	88	48.77	0.28	0.88
20	ined.	gridded records	Malta	39	156	7	19.21	0.01	0.07
21	ined.	gridded records	Montenegro	69	731	25	33.99	0.06	0.25
22	ined.	gridded records	Sicily	95	4203	345	46.8	0.34	3.44
23	Atlas of Flora of Slovenia	atlas	Slovenia	87	2351	33	42.86	0.19	0.33

We indicated for each source the number of taxa (Nb taxa), the number of records (Nb rec), and the number of 10 × 10 km grid cells covered by the source (Nb cells), as well as its contribution to the total WOODIV database (percentage of the taxa (% taxa), of the records (% rec) and of the grid cells (% cells)). Origin and citation of the sources are indicated in Supplementary Table 1 (Ref = reference number to Supplementary Table 1).

When considering the subspecies level, the WOODIV database lacks the occurrences of 11 sub-species among the 35 listed by Médail *et al*.^1^. When aggregated at the species level (to match the taxonomic resolution of the functional and phylogenetic data which are available at the species level only), the WOODIV database lacks only the occurrences of 3 of the 210 species from the Médail *et al*.^1^ checklist (n = 207; Table 2; Supplementary Table 2): *Pyrus elaeagrifolia* Pall., which occurs in Albania and Macedonia (and in northeastern Greece but outside the Mediterranean biome), *P. syriaca* Boiss. and *Tamarix passerinoides* Desv., which occur in Cyprus and in Sardinia, respectively.

**Table 2:** Summary of the availability of data in the WOODIV database: total number of species among the 210 species from the Médail *et al*.^1^ checklist with (1) observed occurrences; (2) functional traits data, including the detail of the number of species with available data for 4 traits: adult plant height (Height), seed mass (SeedMass), specific leaf area (SLA) and wood density (SSD) (see “Functional data” section); and, (3) genetic data including the detail of the number of species with available data for 3 DNA-regions: matK, rbcL and psbA-trnH (see “Genetic data” section).

OBSERVED OCCURRENCES	FUNCTIONAL TRAITS				DNA-REGION SEQUENCES		
207 (**Sp. Agg.** 203)	**Height**	**SeedMass**	**SLA**	**StemSpecDens**	**rbcL**	**trnH**	**matK**
	201	159	102	114	199	195	195
	Total number of species with at least 1 functional trait data: 204				Total number of species with at least 1 DNA-region sequence data: 204		

Sp. Agg. = number of species with observed occurrences when considering the aggregation of *Pinus uncinata* and *P. mugo* into *P. mugo aggr*., *Juniperus deltoides* and *J. oxycedrus* into *J. oxycedrus aggr*., and *Alnus lusitanica*, *A. rohlenae*, and *A. glutinosa* into *A. glutinosa aggr*. (7 species aggregated into 3). The availability of each category of data is detailed by species in Supplementary Table 2.

Also, due to the taxonomic heterogeneity of the different data sources, we recommend aggregating the occurrences of certain tree taxa at the species’ group level (see sections Data Records and Usage Notes): *i.e*. to aggregate *Pinus uncinata* DC. and *P. mugo* Turra into *P. mugo aggr*., *Juniperus deltoides* R.P.Adams and *J. oxycedrus* L. into *J. oxycedrus aggr*. and *Alnus lusitanica* Vít, Douda & Mandák., *A. rohlenae* Vít, Douda & Mandák, and *A. glutinosa* (L.) Gaertn. into *A. glutinosa aggr*. The WOODIV database thus contains reliable occurrences of 200 species and three aggregated species (n = 203; Table 2; Supplementary Table 2).

The raw dataset obtained from gathering occurrences from all sources included a total of 1,248,701 occurrence records distributed across the participating countries.

**Fig. 1:**
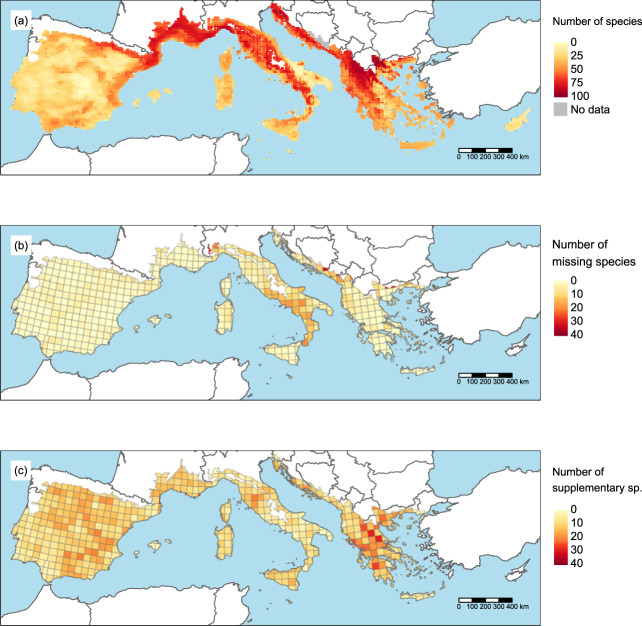
Geographic scope of the WOODIV database, spatial distribution, and validation of trees occurrences. (**a**) Number of species within a 10 × 10 km grid cell based on modelled occurrence data for the 171 modelled species, with the addition of the occurrence data of the 21 small-range species; and, within grid cells of Atlas Flora Europaeae (AFE; 50x50km) (**b**) Number of species with presences recorded in AFE but not in the WOODIV dataset on the 104 species present both in the AFE and WOODIV data; and, (**c**) Number of species with presences recorded in the WOODIV dataset but not in AFE on the 104 species present both in the AFE and WOODIV data.

The raw occurrence data were aggregated at a resolution of 10 × 10 km in line with an INSPIRE^14^ compliant 10 × 10 km grid (SCR 4258). This gridding procedure provided a way to standardize data from different sources. We selected this spatial grain because it was the finest resolution available for some countries of the study area (*e.g*. Slovenia, Croatia, Greece). Sources of occurrence data with a resolution coarser than 10 × 10 km (*e.g*. Atlas Florae Europaeae^15^) were not considered. The considered area includes 10,042 grid cells with at least one occurrence record (Fig. 1a). The occurrence dataset provided by the WOODIV database, *i.e*. aggregated records for species considered as native in the given grid cell using the 10 × 10 km grid (removal of duplicate species within a grid cell) includes 140,279 occurrences.

### Modelled occurrence data

The WOODIV database provides modelled occurrences of the species from the Médail *et al*.^1^ checklist. From the 10 × 10 km gridded observed occurrence data, we modelled the distribution of each species across the Euro-Mediterranean area using Species Distribution Models (SDM). SDM statistically relate species occurrence records to environmental variables to predict the potential distribution of species^16^.

Due to the extent of the study area, we only related species occurrence to climate gradients^17^. Bioclimatic variables were extracted from the CHELSA database V1.2^18^ available at a resolution of 30 arc‐sec (http://chelsa‐climate.org/) and then averaged to a 10 × 10 km resolution. The selection of the environmental predictors for niche modeling is a source of uncertainty in model predictions that can be reduced with sound statistical methods and ecological knowledge of the target species^19^. We also focused on proximal predictors that directly influence species distribution and selected a low number of predictive variables to reduce the issues of model overfitting and multicollinearity^20^. We selected four bioclimatic variables that previous studies had reported to be relevant predictors of the distribution of plant species, especially in environments such as those that characterize the Mediterranean Basin^21–24^: “Minimum temperature of the coldest month” (Bio06, in °C) quantifies potentially lethal frost events and more generally, stress due to low temperatures; “Total annual precipitation” (Bio12, in mm) approximates average water availability; “Precipitation of the driest month” (Bio14, in mm) describes the extremes associated with drought events and stress due to low water availability, and “Temperature seasonality” (Bio04, no dimension) describes the variability of temperature during the year. All selected predictors showed VIF (variance inflation factor^25^) values below 5, indicating that a given predictor was not correlated with any linear combinations of the other predictors (VIF Bio04 = 1.68, VIF Bio06 = 2.06, VIF Bio12 = 1.53, and VIF Bio14 = 2.07).

We related species occurrence to these four bioclimatic variables using the Random Forest algorithm^26^. As only presence data are archived in the WOODIV database, we randomly sampled a number of pseudo-absences equal to the number of observed occurrences^27^. This random selection of pseudo-absences was repeated 10 times for each species. When comparing the floras, occurrence data in the Italian Peninsula, Sardinia and/or Sicily were highly unrepresentative of the distribution of some species (n = 84; see Supplementary Table 3). To overcome this potential bias in the models, we did not include these regions in the model calibration step (Supplementary Table 3). The model was projected in these areas after having tested the similarity in the variables between the projection dataset (Italy, Sicily, and Sardinia) and the fitting dataset (the rest of the study area). Indeed, when model predictions are projected into regions not analyzed in the fitting data, it is necessary to measure the similarity between the new environments and those in the training sample^28^, as models are not so reliable when predicting outside their domain^29^. Similarity analyses computed using ExDet^30^ indicated that all covariables in the projected area are within the univariate range of the fitting area and that there is no change in correlation between covariables (NT1 and NT2 = 0).

Each of these 10 datasets (per species) was then randomly split into two datasets to evaluate model performance on pseudo-independent data^31^: 70% of the data was used to calibrate models and the 30% remaining data was used to evaluate model performance using the True Skill Statistic (TSS^32^) and the Area Under the Curve (AUC) of the receiver-operating characteristic (ROC) plot^33^ metrics. This split-sample step was repeated 10 times resulting in 100 models per species.

For each of the 171 modelled species, a mean model (from the 100 replicates) was then used to predict potential species distribution. Predicted probabilities of occurrence were finally converted into presence/absence using the threshold maximizing the TSS. We fitted all models under the R environment R Core team^34^ and the package biomod2^35,36^.

The WOODIV database provides modelled occurrences of each of the 171 species for each 10 × 10 km grid cell (Fig. 1a). Thirty-two species with less than 10 occurrence records were not modelled (Supplementary Table 3). Among these 32 species, 21 are small-ranged species whose distribution is limited to a few grid cells (Supplementary Table 3). The observed occurrence records for these 21 species can be considered as representative of their distribution and we therefore recommend using the non-modelled records for these species for analyses. The occurrences of the remaining 11 species should be considered unrepresentative of their distribution.

### Functional data

Four functional traits were considered in this project: adult plant height (Height), seed mass (SeedMass), specific leaf area (SLA), and wood density (StemSpecDens). These traits have been proposed to reflect a global spectrum of plant strategies^37,38^: height is a commonly measured proxy for individual size and reflects several aspects including resource acquisition, competitive ability, or dispersal capacity. SeedMass represents the trade-off between fecundity, seed survival, and dispersal. SLA (the ratio between leaf area and dry mass) is correlated to photosynthetic capacity and leaf life span and is an indirect measure of the return on investments in carbon gain compared to water loss. StemSpecDens is a key component of woody plant growth linked to the mechanical support of the stem and its growth rate.

We compiled the values for these traits at the species level for the trees from the Médail *et al*.^1^ checklist, referring mostly to 2 databases: TRY^9^ and BROT 2.0^39^. Supplementary values were obtained from more specific databases (Global Wood Density Database^40^, Kew Seed Information Database^41^) or from the scientific literature and atlas^42–61^. In total, 92% of the entries were extracted from TRY, 7% from BROT 2.0 and the remaining were retrieved from the other sources. The original ID of records from the TRY and BROT databases is provided in order to make it possible to refer to the complete observation if a user needs to have some contextual information.

The WOODIV database lacks all traits data for only 6 of the 210 species from the checklist (Table 2, Supplementary Table 2): *Alnus lusitanica* Vít, Douda & Mandák, *Alnus rohlenae* Vít, Douda & Mandák, *Malus dasyphylla Borkh*., *Quercus infectoria* Olivier, *Tamarix arborea* Ehrenb. ex Bunge and, *Tamarix passerinoides* Del. ex Desf.

**Fig. 2:**
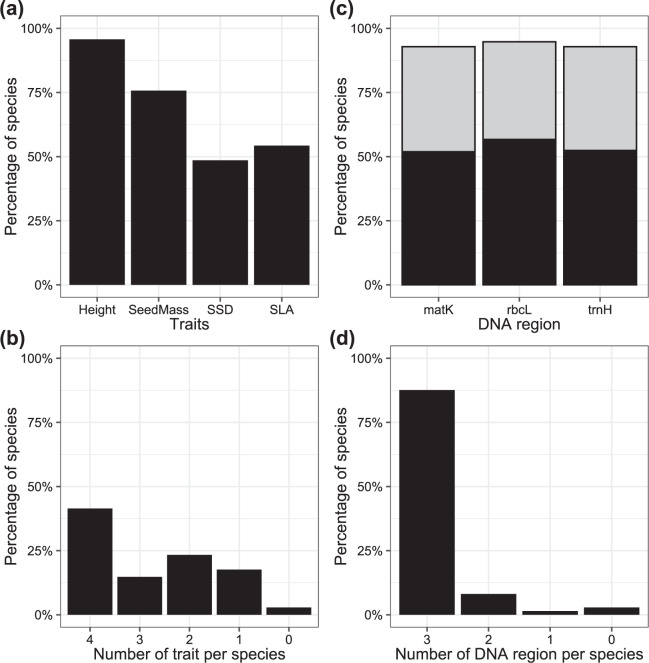
Prevalence of traits and genetic data among the 210 species from Médail *et al*.^1^ checkist: (**a**) For each of the four considered functional traits (adult plant height (Height), seed mass (SeedMass), wood density (SSD) and specific leaf area (SLA)), percentage of the 210 species with existing data; (**b**) Percentage of the 210 species for which none to four functional traits data are available; (**c**) For each of the three considered DNA regions (matK, rbcL and psbA-trnH), percentage of the 210 species with existing data (in grey species with only one available sequence for the considered region, in black species with consensus sequence for that region); and, (**d**) Percentage of the 210 species for which none to three DNA regions data are available.

Adult plant height and seed mass data were available for more than 75% of the 210 species (Table 2; Fig. 2a), whereas wood density and specific leaf area were available for only around 50%. The WOODIV database includes all four trait values for 41% of the 210 species (Fig. 2b; Supplementary Table 2), three trait values for 56% more species.

The database provides an R script that can be used to estimate missing trait values using the taxonomic classification if needed.

### Genetic data

Three different DNA regions from the plastid genome corresponding to the most commonly used DNA barcode regions^62–64^ were considered in this project: the ribulose-bisphosphate/carboxylase Large-subunit gene (*rbc*L), the maturase-K gene (*mat*K), and the *psb*A-*trn*H intergenic spacer (*trn*H).

In a first step, we collected all sequences from GenBank (https://www.ncbi.nlm.nih.gov/genbank/) for the three DNA regions available for the species from the Médail *et al*.^1^ checklist at the species level: *rbc*L: n = 650 sequences for 146 species, *mat*K: n = 644 sequences for 127 species, *trn*H: n = 493 sequences for 129 species). To fill the gaps, we obtained DNA from fresh samples collected in the field or gathered from herbarium specimens (Supplementary Table 4). DNA extraction and sequencing were performed at INRA-URFM, Avignon (France) and the National Research Council (IBBR-CNR), Florence (Italy) (*rbc*L: n = 233 for 125 species, *mat*K: n = 162 for 91 species, *trn*H: n = 200 for 120 species). Methods used for DNA isolation and Sanger sequencing are described by Albassatneh *et al*.^65^. When more than one sequence was available for a given DNA region/species, a sequence alignment was performed to check data quality and a taxon-consensus sequence was generated. Consensus sequences were built using the IUPAC-IUB ambiguity^66^ code for a total of 119 (*rbc*L), 109 (*mat*K), and 110 species (*trn*H), respectively (Fig. 2c). All newly created sequences were uploaded to GenBank.

The WOODIV database lacks the DNA-region sequences data of only 6 of the 210 species from the Médail *et al*.^1^ checklist (Table 2, Fig. 2d): *Alnus lusitanica* Vít, Douda & Mandák, *Cytisus aeolicus* Guss., *Celtis planchoniana* K.I. Chr., *Salix appendiculata* Vill., *Tamarix hampeana* Boiss. & Heldr. and, *Tamarix minoa* J.L. Villar, Turland, Juan, Gaskin, M.A. Alonso & M.B. Crespo.

### Phylogeny

The WOODIV database provides a phylogram including the 204 species for which at least one piece of DNA-region sequence data was available (Supplementary Table 2) and phylograms including the 210 species from the Medail *et al*.^1^ list (Supplementary Fig. 1).

Uneven taxon sampling focused on a single biogeographic area such as ours, can bias phylogenetic inferences^67^. Our goal here is to provide DNA sequence data that can be readily re-used to estimate, e.g. comparable phylogenetic diversity indices, not phylogenetic inferences per se. To illustrate our DNA-sequences data and to facilitate their use for future analyses (to calculate phylogenetic diversity for example), we constructed a molecular phylogeny encompassing the 204 Euro-Mediterranean tree species. Each gene was independently aligned using the MAFFT program^68^ and parsed using the program Gblocks^69^ to exclude the segments characterized by several variable positions or gaps from final alignments. An appropriate substitution model of sequence evolution was selected for each of the three plastid DNA regions using the Akaike Information Criterion (AIC) as implemented in the JModeltest 2 program^70^. The optimal substitution model identified was the same for all three sequences: GTR + I + G. We obtained a concatenated matrix with 1615 aligned bases. We used the Maximum Likelihood analysis^71^ as implemented in the RAxML V8 program^72^. The DNA sequence matrix of 1615 sites was analyzed using three partitions with the GTRGAMMAI model (GTR + Gamma substitution model + proportion of invariant sites). We searched for the optimal tree, running at least 20 independent maximum likelihood analyses; full analyses also consisted of 100 bootstrap replicates^72^.

For users who would like to work on the complete pool of 210 tree species, we also built a 210 species phylogram including all Euro-Mediterranean trees. The six missing species for which no DNA-region sequence was available were added to the phylogenetic tree using the Simulation with Uncertainty for Phylogenetic Investigating (SUNPLIN) method^73^, with 100 replicates. The geometric median tree was computed from the set of 100 replicates with the medTree function from the R package treespace^74^. Both the median tree and the set of 100 replicates are provided in the WOODIV database, together with the molecular tree with 204 species.

## Data Records

**Fig. 3:**
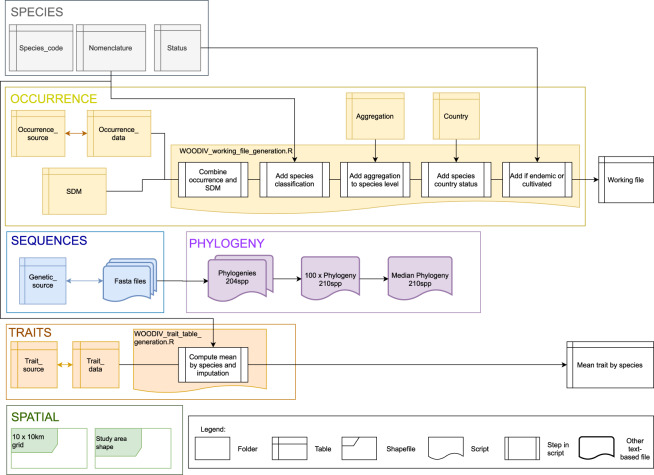
Structure of the WOODIV database. Content of the six folders provided within the database and their links (indicated with arrows), together with the description of the steps included in the two scripts provided (WOODIV_working_file_generation.R and WOODIV_trait_table_generation.R). Occurrence data and the associated information and script are in the yellow box, nomenclature information in the grey box, DNA-region sequences data in the blue box, phylogenetic data in the purple box, the functional data and associated script in the dark orange box, and spatial data files in the dark green box. Contents of provided files are described in Online-only Tables 1 and 2.

The data are available on the *figshare* data repository 10.6084/m9.figshare.13952897.v2^75^ and are comprised of twenty files and two R scripts divided into six folders (Fig. 3), all named following the pattern “*WOODIV_filename.ext*”.

The “SPECIES” folder includes three datasets in comma-separated values (csv) format (Online-only Tables 1, 2): the “*Species_code*” file matches the species code used in the WOODIV database and the scientific name as defined by Médail *et al*.^1^; the “*Nomenclature*” file includes the nomenclature data of all taxa, from the order to the species or subspecies level, and synonymous names if any; the “*Status*” file indicates which taxon is endemic or cultivated following the Médail *et al*.^1^ definition (see Methods section).

The “OCCURRENCE” folder includes five datasets in csv format and one R script (Online-only Tables 1, 2). The “*Occurrence_data*” file includes all observed occurrences of species at the grain size of 10 × 10 km aligned with the INSPIRE LAEA grid, the associated country, and the code of the source from where the data was extracted; the “*SDM*” file includes the modelled occurrences of the (171) species at the 10 × 10 km grid cell-size aligned with the INSPIRE LAEA reference grid; the “*Occurrence_source*” file matches the source code to the full description of the source; the “*Aggregation*” file indicates if taxa can be merged (*e.g*. collapsing all subspecies level data to the species or species’ group level); the “*Country*” file shows whether the taxon is present (native or introduced) or absent in each country; the “*working_file_generation*” R script combines all these datasets into a global dataset.

The “TRAITS” folder includes two datasets in csv format and one R script (Online-only Tables 1, 2): the “*Trait_data*” table includes the functional trait values, the code of the source from where they were extracted, and, when relevant, the source database from which the data is extracted, as well as the ID within this database; the “*Trait_source*” table matches the source code to the full description of the source; the “*trait_table_generation*” R script provides the method to average the trait values at the species level and to replace the missing values with the mean trait values of the higher taxonomic level while recording this level used in a table. The Supplementary Table 5 indicates for each species/trait pair, at which level the value of the trait has been assessed with the current data and code implemented.

The “SEQUENCES” folder includes one dataset in csv format and three text-based files for representing nucleotide sequences (FASTA). The “*Sequence_source*” file shows the GenBank reference number of each DNA-region sequence together with the data source (either GenBank or WOODIV); the sources of the samples sequenced by the WOODIV consortium are listed in Supplementary Table 4; the fasta files refer to the sequences (unique or consensus) for each species and DNA-region used to build the phylogenetic tree of the 204 species and named according to the DNA-region.

The “PHYLOGENY” folder includes four phylogenetic trees. The “*Phylogeny_204spp_BS*” file includes the phylogram of the 204 species for which at least one of the three DNA-region sequences was available with bootstrap values, in nexus format. The “*Phylogeny_204spp*” file includes the same phylogram without bootstrap in Newick format. The “*Phylogenies_210spp_100rep*” file includes the 100 replicates of the phylogeny of the 210 species from the checklist, in Newick format. The “*Phylogeny_210spp_median*” file includes the median tree from the 100 replicates for the 210 species, in Newick format.

The “SPATIAL” folder has two subfolders: the “*Study_area_shape*” subfolder includes a polygon shapefile (EPSG: 3035) delimitating the study area while the “*10 × 10 km_grid*” subfolder includes a polygon shapefile (EPSG: 3035) displaying the part of the INSPIRE LAEA grid that covers the study area.

## Technical Validation

### Observed and modelled occurrence data

The first step of data validation when gathering occurrence data is to agree on a taxonomic backbone. We followed the list of accepted names and their synonyms compiled by Médail *et al*.^1^ for all Euro-Mediterranean tree species. The WOODIV database includes a taxonomy table which provides the nomenclature from different taxonomic references: EURO + MED Plant Base (http://www.emplantbase.org/home.html), the Browicz^76^, and the World Checklist Kew (http://wcsp.science.kew.org).

Errors in georeferenced data are common, but many of them can be easily detected^77^. We systematically filtered the data to discard records (i) with missing latitude or longitude or (ii) falling outside the study area covered by the data source (*e.g*. outside the borders of a country for a national atlas), and standardized the projection system if needed. In other cases, coordinates for species records appear correct but could fall outside the known and validated range of a species, mostly due to uncertain or erroneous taxonomic identification. These cases are more complicated to detect, requiring validation by an expert and/or comparison with an independent dataset to distinguish a false identification from a validated location. This step is often neglected due to lack of time or because the expertise is not available. In the WOODIV project, we implemented these two time-consuming validation steps: (i) using independent data provided at the country level to discard records falling outside the known species range. This step was led by the botanists using the country checklist of trees in Mediterranean Europe published by the same authors^1^. For Spain only, botanists also compared the spatial distribution of occurrences available from the GBIF platform with the occurrences maps provided by Flora Iberica^61^ and Flora-On (https://flora-on.pt/); (ii) checking the resulting maps of occurrences to discard dubious records by botanists from each of the 13 countries and islands. This validation step, for example, resulted in the deletion of records of planted trees such as those of *Abies pinsapo* Boiss. planted outside the native range in southern Spain.

To assess gaps in the occurrence data within the WOODIV dataset, we compared our occurrence data with the data of the Atlas Flora Europaeae (AFE)^15^. The AFE provides the distribution data for vascular plants in Europe at a 50 × 50 km resolution. We compared the occurrence distribution only for species in both the AFE and the WOODIV data (n = 104). For each of these species, we checked whether our dataset provides occurrence in the grid cells where the AFE reported presence. AFE grid cells where occurrences are missing in our dataset (Fig. 1b) and where our dataset reports occurrence data when the AFE does not (Fig. 1c) were mapped. Overall, the comparison with AFE (on 10,585 occurrences in the 50 × 50 km grid cells in AFE, for the 104 species) showed that we brought more occurrence information (n = 5405, i.e. + 51.1%) than we missed (n = 2186, i.e. 20.7%), suggesting the strong input of our database for Euro-Mediterranean trees. The most important gaps in the data occurred in Italy and in Montenegro (inland as we collected additional data on the field in the coastal area).

All species distribution models were tested for their predictive ability on the evaluation dataset using both the AUC and the TSS metric (Supplementary Table 3). A filter was applied to modelled occurrences based on the presence or not of species in each country as indicated by Médail *et al*.^1^ Thus, modelled but unconfirmed occurrences of species, namely “false occurrences” were converted to absences.

### Trait data

Datasets from juvenile stages were systematically discarded. Trait measures were checked for consistency in the unit (m for Height, mg for SeedMass, g.cm^−3^ for StemSpecDens, and mm^2^.mg^−1^ for SLA). Categorical coded values (*e.g*. high, medium, heavy) and extreme outliers were removed. For species with shrub and tree forms, maximum or range values of Height were taken only for tree forms. When coordinates were provided in the databases, we filtered out those from outside of the Euro-Mediterranean Basin in order to keep trait variation observed within the region. Finally, redundancies between the different sources were checked and duplicated entries were removed to keep only one entry.

### Genetic data and phylogeny

For each taxon, sequences were quality checked and edited using CodonCode Aligner (CondonCode Co., MA, USA) to trim and remove low-quality regions. For sequences from GenBank, long sequences were preferred. For INRA-URFM and IBBR-CNR sequences, the quality of the chromatograms was visually checked, and ambiguous nucleotides were called using the uncertainty code. All sequences were blasted and matched with the closest relatives. Sequences falling outside genus sections were removed from the data set. Multiple sequence alignments were built using the program MAFFT^42^ and parsed using the program Gblocks^43^ to exclude the segments characterized by several variable positions or gaps from final alignments. The monophyly of families and genera was checked in the inferred phylogeny. In case of non-monophyly, the sequences were blasted again to validate them. We compared the topology of orders and above in our tree with the tree published in APGIV to make sure that the topologies were mainly congruent. The slight discrepancies we observe with reference phylogenies are mostly in families that are notoriously phylogenetically complicated, with incomplete lineage sorting and frequent speciation events, as in the Rosaceae and the Fagaceae.

## Usage Notes

Two summary tables can be generated from the different tables of the WOODIV database following the workflow presented in Fig. 3, using the scripts included in the database. The first table, named “*working file*”, is generated by the “*WOODIV_working_file_generation.R*” script, which relates all information regarding the species and occurrences. As a first step, the observed recorded (“*Occurrence_data*”) and the modelled (“*SDM*”) occurrences are merged into one, with a variable indicating the type of data for each occurrence (observed or modelled). Then the classification of each species (“*Nomenclature*” data) is added to the table, using the species code as an index. The next step inserts the information about taxa aggregation (“*Aggregation*” data) at the species or species’ group level as described in the “Methods” section. The status of the species in each country (native or introduced) is added for each occurrence using the “*Country*” data. The last information added to the table is the cultivated or endemic status of the species (“*Status*” data). Other variables (*e.g*. the scientific name of each species for each occurrence) or filters (*e.g*. to select only the SDM outputs) can be easily generated from this resulting table (“*working file*”).

The second table includes a summary of the functional traits for each species. The “*WOODIV_trait_table_generation.R*” script can be used to compute the mean value for each trait and each species (“*Mean trait by species*” table) from all trait measures included in the “*Trait_data*”. In addition, the “*Nomenclature*” data can be used to impute values for species with no value for a given trait based on the taxonomic classification by taking the mean values of higher rank. Genus, family, or order levels are currently implemented in the script.

The “*working file*” table, the “*mean trait by species*” table, the 210-species phylogenetic tree, and the spatial layers are organized to easily perform several analyses, as diversity maps: the “*working file*” table can be filtered to keep either the observed or the modelled occurrences and converted into a community matrix giving the number of occurrences of each taxa in each cell, using the cell ID as row and the aggregation level as column. Diversity metrics can be derived from this matrix (*e.g*. the number of occurrences or of taxa by cell), as well as phylogenetic and functional metrics using appropriate tools and functions. To derive the latter, the match and ranking between the taxa labels of the occurrences, traits, and phylogenetic data must be carefully checked (*e.g*. using the organize.syncsa function in the SYNCSA R package or the match.function groups in the PICANTE R package). Maps can then be generated using the cell grid layer to spatialize the metrics.

As biodiversity data are rapidly accumulating, new information will become available. The same standardized cleaning and filtering processes can be applied to upcoming occurrences and traits data, and the future updates of the database will be uploaded as new versions of the database on the same *figshare* data repository^75^ once a year. If a user has an error to report or a suggestion to improve the database, the corresponding author can be contacted.

## Supplementary information

Supplementary Information

## Data Availability

Two R scripts are available with the data files in the database. The “*WOODIV_working_file_generation.R*” script in the “SPECIES” folder combines all the information about species occurrences and nomenclature into one table to run the analyses. The “*WOODIV_trait_table_generation.R*” in the “TRAITS” folder uses the species nomenclature to compute species mean traits and impute values when no data is available using nomenclature. They run under R software version 3.6 (last tests under version 3.6.2^34^).

## Online-only Tables

**Online-only Table 1:** Description of the data included in the WOODIV database and the name of the corresponding file(s).

	Data name	Data description	Data file name
SPECIES	Species_code	This table matches the species indexation code to the full name of species	WOODIV_Species_code.csv
	Nomenclature	This table includes the taxonomic assignation of each species from the class to the subspecies level, and if the species has synonyms in Euro + Med PlantBase, Browicz or Kew databases.	WOODIV_Nomenclature.csv
	Status	This table indicates the endemism status of each species and if it is native, introduced, cultivated or not.	WOODIV_Status.csv
OCCURRENCE	Occurrence_data	This table includes the occurrences of species in the LAEA grid and from where the information was extracted.	WOODIV_Occurrence_data.csv
	Occurrence_source	This table matches the occurrence source code to the source from where occurrences where extracted from.	WOODIV_Occurrence_source.csv
	SDM	This table includes the modelled of species in the LAEA grid	WOODIV_SDM.csv
	Aggregation	This table indicates the subspecies level data which can be aggregated at the species level and the species level data which can be pooled together.	WOODIV_Aggregation.csv
	Country	This table indicates the occurrence status of species at the country level	WOODIV_Country.csv
TRAITS	Trait_data	This table includes the individual trait values for the four traits extracted from trait databases.	WOODIV_Trait_data.csv
	Trait_source	This table matches the trait source code to the source from where trait values were extracted.	WOODIV_Trait_source.csv
SEQUENCES	Sequence_data	These files include the unique or consensus (if more than one sequence was available for the species) sequence per species for each DNA-region used to build the phylogenetic tree of the 204 species.	WOODIV_matK.fasta; WOODIV_rcbL.fasta; WOODIV_trnH-psbA.fasta
	Sequence_source	This table includes the reference numbers in GenBank of the DNA-regions sequences of the database, together with the source of the data.	WOODIV_Sequence_source.csv
PHYLOGENY	Phylogenies 204spp	These files include the phylogram of the 204 species for which at least one of the three DNA-region sequences was available with bootstrap values, in nexus format, and without bootstrap values, in newick format.	WOODIV_Phylogeny_204spp_BS.nexus; WOODIV_Phylogeny_204spp.tree
	100 x Phylogeny 210spp	This file includes the 100 phylograms (replicates) including the 210 species from the checklist of Médail *et al*.^1^, in newick format	WOODIV_Phylogenies_210spp_100rep.tree
	Median Phylogeny 210spp	This file includes the median phylogram of the 100 replicates including the 210 species from the checklist of Médail *et al*.^1^ in newick format	WOODIV_Phylogeny_210spp_median.tree
SPATIAL	10 × 10 km grid	This folder includes the extracted part of the INPIRE LAEA grid (10 × 10 km) that covers the study area as polygon shapefile.	WOODIV_grid/
	Study area shape	This folder includes the study area as polygon shapefile.	WOODIV_shape/

**Online-only Table 2:** Description of the columns within the files of the WOODIV database.

Data file name	Column name	Column description
Species_code	Spcode	Species indexation code
	genus_species_subspecies	Species full name (or subspecies if any)
Nomenclature	Spcode	Species indexation code
	Class	Taxonomic rank at the Class level
	Subclass	Taxonomic rank at the Subclass level
	Order	Taxonomic rank at the Order level
	Family	Taxonomic rank at the Family level
	Genus	Taxonomic rank at the Genus level
	Species	Taxonomic rank at the Species level
	authority_species	Authority for the Species level
	subspecies	Taxonomic rank at the Subspecies level, if any
	authority_subspecies	Authority for the Subpecies level, if any
	Euro + Med _taxon_name	Synonymy in the Euro + Med PlantBase
	Browicz_taxon_name	Synonymy in the Browicz database
	Kew_taxon_name	Synonymy in the Kew database
Status	spcode	Species indexation code
	endemism	Is the species endemic of…: 0/ no, 1/yes
	cultivated	Is the species cultivated: 0/no, 1/yes
Occurrence_data	spcode	Species indexation code
	Idgrid	Cell identity in the LAEA grid
	source_code	Code of the occurrence source
	country	Countries and islands considered in the WOODIV database: Albania, Balearic, Corsica, Crete, Croatia, Cyprus, France, Greece, Italy, Macedonia, Malta, Montenegro, Portugal, Sardinia, Sicily, Slovenia, Spain
Occurrence_source	source_code	Code of the occurrence source
	source_country	Countries for which the source provided data
	source_name	Name of the source of data
	data_type	Is the data observations or atlas data
	source_citation	Citation and acknowledge to the data providers
SDM	spcode	Species indexation code
	Idgrid	Cell identity in the LAEA grid
	country	Countries and islands considered in the WOODIV database: Albania, Balearic, Corsica, Crete, Croatia, Cyprus, France, Greece, Italy, Macedonia, Malta, Montenegro, Portugal, Sardinia, Sicily, Slovenia, Spain
Aggregation	spcode	Species indexation code
	to_aggregate_with	In cases of aggregation of data between species, indicates which species they must be aggregated with. For subspecies, it indicates the corresponding species level.
Country	spcode	Species indexation code
	country	Countries and islands considered in the WOODIV database: Albania, Balearic, Corsica, Crete, Croatia, Cyprus, France, Greece, Italy, Southern Macedonia, Malta, Montenegro, Portugal, Sardinia, Sicily, Slovenia, Spain
	status	Occurrence status of the species at the country level: A/ absent, I/ introduced, N/ native, N?/ putative native,?/ Unknown
Trait_data	spcode	Species indexation code
	Traits	Traits code: Height/ adult plant height, SeedMass/ seed mass, SLA/ specific leaf area, StemSpecDens/ wood density
	Value	The value reported for the trait
	Trait_source	Code of the source from where the value was extracted
	valueID_in_database	ID of the value when extracted from a database (NA otherwise)
Trait_source	Trait_source	Code of the source from where the value was extracted
	Source	Source from where the value was extracted
Sequence_source	spcode	Species indexation code
	DNA_region	DNA-region to which the sequence refers (either *rbc*L, *mat*K or *trn*H)
	Accession_number	Sequence accession number in GenBank
	Source	Source of the sequence: GB = sequence obtained from GenBank, WD = new data created by the WOODIV consortium and uploaded into GenBank
