# Author Correction: WOODIV, a database of occurrences, functional traits, and phylogenetic data for all Euro-Mediterranean trees

**DOI:** 10.1038/s41597-021-00911-0

**Published:** 2021-05-04

**Authors:** Anne-Christine Monnet, Kévin Cilleros, Frédéric Médail, Marwan Cheikh Albassatneh, Juan Arroyo, Gianluigi Bacchetta, Francesca Bagnoli, Zoltán Barina, Manuel Cartereau, Nicolas Casajus, Panayotis Dimopoulos, Gianniantonio Domina, Aggeliki Doxa, Marcial Escudero, Bruno Fady, Arndt Hampe, Vlado Matevski, Stephen Misfud, Toni Nikolic, Daniel Pavon, Anne Roig, Estefania Santos Barea, Ilaria Spanu, Arne Strid, Giovanni Giuseppe Vendramin, Agathe Leriche

**Affiliations:** 1 Aix Marseille Univ, Avignon Univ, CNRS, IRD, IMBE. Technopôle de l’Arbois-Méditerranée, cedex 4, BP 80, 13 545 Aix-en-Provence, France; 2 Institut de Systématique, Evolution, Biodiversité (ISYEB), Muséum national d’Histoire naturelle (MNHN), CNRS, Sorbonne Université, EPHE, Université des Antilles, Paris, France; 3 Department of Plant Biology and Ecology, University of Seville, Seville, Spain; 4 Department of Life and Environmental Sciences, University of Cagliari, Viale Sant’Ignazio da Laconi 13, Cagliari, Italy; 5 National Research Council, Institute of Biosciences and Bioresources, 50019 Sesto Fiorentino, (FI) Italy; 6 Department of Botany, Hungarian Natural History Museum, Pf. 137, Budapest, 1431 Hungary; 7 FRB-CESAB, 5 rue de l’Ecole de Médecine, 34000 Montpellier, France; 8 Department of Biology, Laboratory of Botany, University of Patras, 26504 Patras, Greece; 9 Department of Agriculture, Food and Forest Sciences, University of Palermo, Viale delle Scienze bldg. 4, 90128 Palermo, Italy; 10 Statistical Learning Lab, Institute of Applied and Computational Mathematics, Foundation for Research and Technology-Hellas (FORTH), Ν. Plastira 100, Vassilika Vouton, GR - 700 13 Heraklion, Crete Greece; 11 INRAE, UR629, Ecologie des forêts méditerranéennes, Avignon, France; 12 INRAE, Univ. Bordeaux, BIOGECO, F-33610 Cestas France; 13 Macedonian Academy of Sciences and Arts, Krste Misirkov 2, 1000 Skopje, Republic of Macedonia; 14 EcoGozo, Regional Development Directorate - Ministry for Gozo, Flat 6, Sunset Court B, Triq Marsalforn, Xaghra, Gozo Malta; 15 Department of Botany, Faculty of Science, University of Zagreb, Zagreb, Croatia; 16 Bakkevej 6, 5853 Ørbæk, Denmark

**Keywords:** Conservation biology, Community ecology, Biodiversity, Forest ecology, Biogeography

Correction to: *Scientific Data* 10.1038/s41597-021-00873-3, published online 23 March 2021

The original version of this Data Descriptor contained an error in the author affiliations. Marwan Cheikh Albassatneh was incorrectly associated with *Institute of Ecology and Environmental Sciences, Sorbonne University, Paris, France* and *Institut de Systématique, Evolution, Biodiversité (ISYEB), Muséum national d'Histoire naturelle (MNHN), CNRS, Sorbonne Université, EPHE, Université des Antilles, Paris, France* was inadvertently omitted. This has now been corrected in both the PDF and HTML versions of the Data Descriptor.

