# Supplementary material for: WOODIV, a database of occurrences, functional traits, and phylogenetic data for all Euro-Mediterranean trees
Source: Sci Data. 2021 Mar 23;8:89. doi: 10.1038/s41597-021-00873-3 (PMC7988049; doi:10.1038/s41597-021-00873-3)

**Supplementary Information**

***Supplementary Table 1. References of the sources of the occurrence records****. Ref is the reference number to Table 1.*

| **Ref** | **Source country** | **Citation** |
| --- | --- | --- |
| 1 | Albania | Barina, Zoltán (2017) Distribution atlas of vascular plants in Albania. Hungarian Natural History Museum, Budapest. 492 pp. |
| 2 | Croatia | Nikolic T. ed. (2015-onward): Flora Croatica Database (URL: https://hirc.botanic.hr/fcd/). Faculty of Science, University of Zagreb (Accessed the 08/09/2016) |
| 3 | Cyprus | Charalambos S. Christodolou, personal communication. Accessed the 27/02/2018 |
| 4 | Europe | Genetic Conservation Units from the EUFGIS (http://portal.eufgis.org) provided by the European Forest Genetic Resources Programme (EUFORGEN) on 04/01/2017 |
| 5 | Cyprus | For Cyprus only, Serra-Diaz et al. (2017) Big data of tree species distributions: how big and how good? For. Ecosyst. 4, 30. Accessed the 20/10/2017 |
| 6 | France | Conservatoire botanique national du Massif central. 14 février 2017 < CHLORIS® système d’information dédié à la flore sauvage et aux végétations du Massif central > |
| 7 | France | Conservatoire botanique national Alpin (CBNA). Accessed the 28/03/2017 |
| 8 | France, Corsica | Conservatoire botanique national de Corse (CBNC). Accessed the 09/11/2017 |
| 9 | France, Corsica | IGN Inventaire forestier (http:/inventaire-forestier.ign.fr). Accessed the 05/04/2016 |
| 10 | France, Corsica | Système d’Information et de Localisation des Espèces Natives et Envahissantes (SILENE) (http://flore.silene.eu). Accessed the 21/10/2016 |
| 11 | Greece, Crete | Flora Hellenica Database (personal communication, prof. A. Strid, 2019)  Dimopoulos P, Raus T, Strid A (ed.) (2018) Flora of Greece web. Vascular Plants of Greece. An Annotated Checklist. Version II (June 2018). Published at: http://portal.cybertaxonomy.org/flora-greece/, accessed 20 August 2018  Dimopoulos P, Raus T, Bergmeier E, et al (2013) Vascular plants of Greece: an annotated checklist. – Berlin: Botanischer Garten und Botanisches Museum Berlin-Dahlem, Freie Universität Berlin, Athens: Hellenic Botanical Society. Englera, 31: 1-370 |
| 12 | Portugal, Spain, Balearic Islands | GBIF.org, Flora Iberica, Castroviejo, S. (coord. gen.). 1986-2012. Flora iberica 1-8, 10-15, 17-18, 21. Real Jardín Botánico, CSIC, Madrid., accessed with Anthos (http://www.anthos.es), Flora-On: Flora de Portugal Interactiva. (2014). Sociedade Portuguesa de Botânica. www.flora-on.pt. |
| 13 | Eolian islands | Pasta S., La Rosa A., Pavon D., Lo Cascio P., Médail F. *Tentamen Florae Aeolicae*: A critical checklist of the vascular plants of the Aeolian Islands (Sicily, Italy). in prep. |
| 14 | Italy | Data from the National Research Council (Italy)  For *Abies alba*: Piotti A., Leonarduzzi C., Postolache D., Bagnoli F., Spanu I., Brousseau L., Urbinati C., Leonardi S., Vendramin G.G. (2017). Unexpected scenarios from Mediterranean refugial areas: disentangling complex demographic dynamics along the Apennine distribution of silver fir. Journal of Biogeography, 44(7), 1547-1558.  For *Fagus sylvatica*: Bagnoli F., Piotti A., Vendramin G.G., personal communication  For *Pinus halepensis*: Gómez A, Vendramin G.G., González-Martínez S.C., Alía R. (2005). Genetic diversity and differentiation of two Mediterranean pines (*Pinus halepensis* Mill. and *Pinus pinaster* Ait.) along a latitudinal cline using chloroplast microsatellite markers. Diversity and Distribution, 11(3): 257-263., Bucci G., Anzidei M., Madaghiele A., Vendramin G.G. (1998). Detection of haplotypic variation and natural hybridization in *P. halepensis*?complex pine species using chloroplast simple sequence repeat (SSR) markers. Molecular Ecology, 7(12): 1633-1643.  For *Pinus heldreichii*: Bagnoli F., Piotti A., Vendramin G.G., personal communication  For *Quercus cerris*: Bagnoli F., Tsuda Y., Fineschi S., Bruschi P., Magri D., Zhelev P., Paule L., Simeone M.C., González-Martínez S.C. & Vendramin G.G. (2016) Combining molecular and fossil data to infer demographic history of *Quercus cerris*: insights on European eastern glacial refugia. Journal of Biogeography, 43, 679-690. |
| 15 | Italy | Mauri, A. et al. 2017. EU-Forest, a high-resolution tree occurrence dataset for Europe. - Sci. Data 4: 160123. |
| 16 | Italy | Terzo inventario forestale nazionale (INFC2015) https://inventarioforestale.org/it |
| 17 | Italy | www.vegitaly.it, Lucarini D., Gigante D., Landucci F., Panfili E., Venanzoni R. 2015. The anArchive taxonomic Checklist for Italian botanical data banking and vegetation analysis: theoretical basis and advantages. Plant Biosyst., 149(6): 958-965. doi: 10.1080/11263504.2014.984010, Gigante D., Acosta A.T.R., Agrillo E., Attorre F., Cambria V.M., Casavecchia S., Chiarucci A., Del Vico E., De Sanctis M., Facioni L., Geri F., Guarino R., S. Landi, Landucci F., Lucarini D., Panfili E., Pesaresi S., Prisco I., Rosati L., Spada F., Venanzoni R., 2012. VegItaly: Technical features, crucial issues and some solutions. Plant Sociology, 49(2): 71-79. doi: 10.7338/pls2012492/05, Landucci F., Acosta A.T.R., Agrillo E., Attorre F., Biondi E., Cambria V.M., Chiarucci A., Del Vico E., De Sanctis M., Facioni L., Geri F., Gigante D., Guarino R., S. Landi, Lucarini D., Panfili E., Pesaresi S., Prisco I., Rosati L., Spada F., Venanzoni R., 2012. VegItaly: The Italian collaborative project for a national vegetation database. Plant Biosyst., 146(4): 756-763. doi: 10.1080/11263504.2012.740093, Venanzoni R., Landucci F., Panfili E., Gigante D., 2012. Toward an Italian national vegetation database: VegItaly. In: Dengler, J., Oldeland, J., Jansen, F., Chytry, M., Ewald, J., Finckh, M., Glöckler, F., Lopez-Gonzalez, G., Peet, R.K., Schaminée, J.H.J. [Eds.]: Vegetation databases for the 21st century. Biodiversity & Ecology, 4: 185-190. ISSN: 1613-9801 |
| 18 | Italy | GBIF.org (21st June 2019) GBIF Occurrence Download https://doi.org/10.15468/dl.jnctii  GBIF.org (21st June 2019) GBIF Occurrence Download https://doi.org/10.15468/dl.5s23qb  GBIF.org (21st June 2019) GBIF Occurrence Download <https://doi.org/10.15468/dl.seopf2>  GBIF.org (27th June 2019) GBIF Occurrence Download https://doi.org/10.15468/dl.fd3wnl |
| 19 | Macedonia | V. Matevski, ined. |
| 20 | Malta | Stephen Mifsud, ined. |
| 21 | Montenegro | Médail & Monnet, ined. |
| 22 | Sicily | Gianniantonio Domina, ined. |
| 23 | Slovenia | N Jogan, T Bacic, B Frajman, I Leskovar, D Naglic, A Podobnik, B Rozman, S Strguljc-Krajšek, B Trcak, 2001. Materials for the Atlas of Flora of Slovenia, Center za kartiranje favne in flore. Miklavz na Dravskem polju |

***Supplementary Table 2. Availability (1 = available) of the different category of data (occurrences, functional traits and DNA-region sequences) in the WOODIV database per species*** *for the 210 species from the checklist with 1) observed occurrences (OBS. OCC.); 2) data for the four functional traits: adult plant height (Height), seed mass (SeedMass), specific leaf area (SLA) and wood density (StemSpecDens); and, 3) genetic data with each considered DNA-region: rbcL, trnH and matK. Sp. Code = species code as in the “Species_code” file into the WOODIV database. Underlined species names correspond to species for which we recommend aggregating the occurrences data under the Sp. Aggr. corresponding taxa (e.g. Alnus lusitanica to be aggregated into A. glutinosa aggr.). NA = Not Available.*

| **Sp. code** | **Species** | **OBS. OCC.** | **FUNCTIONAL TRAITS** | | | | **DNA-REGION SEQUENCES** | | |
| --- | --- | --- | --- | --- | --- | --- | --- | --- | --- |
|  |  |  | **Height** | **SeedMass** | **SLA** | **StemSpecDens** | **rbcL** | **trnH** | **matK** |
| **AALB** | *Abies alba* | 1 | 1 | 1 | 1 | 1 | 1 | 1 | 1 |
| **AAND** | *Arbutus andrachne* | 1 | 1 | 1 | 1 | 1 | 1 | 1 | 1 |
| **ABOR** | *Abies borisii-regis* | 1 | 1 | 1 | NA | NA | 1 | 1 | 1 |
| **ACAM** | *Acer campestre* | 1 | 1 | 1 | 1 | 1 | 1 | 1 | 1 |
| **ACAP** | *Acer cappadocicum* | 1 | 1 | 1 | NA | NA | 1 | 1 | 1 |
| **ACEP** | *Abies cephalonica* | 1 | 1 | 1 | NA | NA | 1 | 1 | 1 |
| **ACOR** | *Alnus cordata* | 1 | 1 | 1 | NA | 1 | 1 | 1 | 1 |
| **AGLU** | ***Alnus glutinosa*** | 1 | 1 | 1 | 1 | 1 | 1 | 1 | 1 |
| **AHEL** | *Acer heldreichii* | 1 | 1 | 1 | NA | NA | 1 | 1 | 1 |
| **AHIP** | *Aesculus hippocastanum* | 1 | 1 | 1 | 1 | 1 | 1 | 1 | 1 |
| **AHYR** | *Acer hyrcanum* | 1 | 1 | 1 | NA | NA | 1 | 1 | 1 |
| **AINC** | *Alnus incana* | 1 | 1 | 1 | 1 | 1 | 1 | 1 | 1 |
| **ALUS** | *Alnus lusitanica* (*A. glutinosa aggr.)* | 1 | NA | NA | NA | NA | NA | NA | NA |
| **AMON** | *Acer monspessulanum* | 1 | 1 | 1 | 1 | 1 | 1 | 1 | 1 |
| **ANEB** | *Abies nebrodensis* | 1 | 1 | NA | 1 | NA | 1 | 1 | 1 |
| **AOBT** | *Acer obtusifolium* | 1 | 1 | 1 | NA | 1 | 1 | 1 | 1 |
| **AOPA** | *Acer opalus* | 1 | 1 | 1 | 1 | 1 | 1 | 1 | 1 |
| **AORI** | *Alnus orientalis* | 1 | 1 | 1 | NA | 1 | 1 | 1 | 1 |
| **APIN** | *Abies pinsapo* | 1 | 1 | 1 | 1 | 1 | 1 | 1 | 1 |
| **APLA** | *Acer platanoides* | 1 | 1 | 1 | 1 | 1 | 1 | 1 | 1 |
| **APSE** | *Acer pseudoplatanus* | 1 | 1 | 1 | 1 | 1 | 1 | 1 | 1 |
| **AROH** | *Alnus rohlenae* (*A. glutinosa* aggr.) | 1 | NA | NA | NA | NA | 1 | 1 | 1 |
| **ASEM** | *Acer sempervirens* | 1 | 1 | 1 | NA | NA | 1 | 1 | 1 |
| **ATAT** | *Acer tataricum* | 1 | 1 | 1 | NA | NA | 1 | 1 | 1 |
| **AUNE** | *Arbutus unedo* | 1 | 1 | 1 | 1 | 1 | 1 | 1 | 1 |
| **AVIR** | *Alnus viridis* | 1 | 1 | 1 | NA | NA | 1 | 1 | 1 |
| **BBAL** | *Buxus balearica* | 1 | 1 | 1 | 1 | NA | 1 | 1 | 1 |
| **BPEN** | *Betula pendula* | 1 | 1 | 1 | 1 | 1 | 1 | 1 | 1 |
| **BPUB** | *Betula pubescens* | 1 | 1 | 1 | 1 | 1 | 1 | 1 | 1 |
| **BSEM** | *Buxus sempervirens* | 1 | 1 | 1 | 1 | 1 | 1 | 1 | 1 |
| **CAEO** | *Cytisus aeolicus* | 1 | 1 | NA | NA | NA | NA | NA | NA |
| **CAOR** | *Carpinus orientalis* | 1 | 1 | 1 | 1 | NA | 1 | 1 | 1 |
| **CAUS** | *Celtis australis* | 1 | 1 | 1 | 1 | 1 | 1 | 1 | 1 |
| **CAVE** | *Corylus avellana* | 1 | 1 | 1 | 1 | 1 | 1 | 1 | 1 |
| **CAZA** | *Crataegus azarolus* | 1 | 1 | 1 | NA | 1 | 1 | 1 | 1 |
| **CBET** | *Carpinus betulus* | 1 | 1 | 1 | 1 | 1 | 1 | 1 | 1 |
| **CCOG** | *Cotinus coggygria* | 1 | 1 | 1 | 1 | 1 | 1 | 1 | 1 |
| **CCOL** | *Corylus colurna* | 1 | 1 | 1 | NA | NA | 1 | 1 | 1 |
| **CGRA** | *Cotoneaster granatensis* | 1 | 1 | 1 | NA | NA | 1 | 1 | 1 |
| **CHEL** | *Crataegus heldreichii* | 1 | 1 | NA | NA | NA | 1 | 1 | 1 |
| **CHUM** | *Chamaerops humilis* | 1 | 1 | 1 | 1 | NA | 1 | 1 | 1 |
| **CLAC** | *Crataegus laciniata* | 1 | 1 | NA | NA | NA | 1 | NA | 1 |
| **CLAE** | *Crataegus laevigata* | 1 | 1 | 1 | 1 | NA | 1 | 1 | 1 |
| **CLIB** | *Cedrus libani* | 1 | 1 | 1 | NA | 1 | 1 | 1 | 1 |
| **CMON** | *Crataegus monogyna* | 1 | 1 | 1 | 1 | 1 | 1 | 1 | 1 |
| **CNEV** | *Crataegus nevadensis* | 1 | 1 | NA | NA | NA | 1 | 1 | 1 |
| **CPEN** | *Crataegus pentagyna* | 1 | 1 | 1 | NA | NA | 1 | 1 | 1 |
| **CPLA** | *Celtis planchoniana* | 1 | 1 | 1 | NA | NA | NA | NA | NA |
| **CPYC** | *Crataegus pycnoloba* | 1 | 1 | NA | NA | NA | NA | 1 | NA |
| **CROR** | *Crataegus orientalis* | 1 | 1 | 1 | NA | NA | 1 | 1 | 1 |
| **CSAT** | *Castanea sativa* | 1 | 1 | 1 | 1 | 1 | 1 | 1 | 1 |
| **CSEM** | *Cupressus sempervirens* | 1 | 1 | 1 | 1 | 1 | 1 | 1 | 1 |
| **CSIA** | *Ceratonia siliqua* | 1 | 1 | 1 | 1 | 1 | 1 | 1 | 1 |
| **CSIM** | *Cercis siliquastrum* | 1 | 1 | 1 | 1 | 1 | 1 | 1 | NA |
| **CTOU** | *Celtis tournefortii* | 1 | 1 | NA | NA | 1 | 1 | 1 | 1 |
| **EARB** | *Erica arborea* | 1 | 1 | 1 | 1 | 1 | 1 | 1 | 1 |
| **FALN** | *Frangula alnus* | 1 | 1 | 1 | 1 | 1 | 1 | 1 | 1 |
| **FANG** | *Fraxinus angustifolia* | 1 | 1 | 1 | 1 | 1 | 1 | 1 | 1 |
| **FCAR** | *Ficus carica* | 1 | 1 | 1 | 1 | 1 | 1 | 1 | 1 |
| **FEXC** | *Fraxinus excelsior* | 1 | 1 | 1 | 1 | 1 | 1 | 1 | 1 |
| **FORI** | *Fagus orientalis* | 1 | 1 | 1 | NA | 1 | 1 | 1 | 1 |
| **FORN** | *Fraxinus ornus* | 1 | 1 | 1 | 1 | 1 | 1 | 1 | 1 |
| **FPAL** | *Fraxinus pallisae* | 1 | 1 | 1 | NA | NA | 1 | 1 | 1 |
| **FPHI** | *Fontanesia philly~~i~~raeoides* | 1 | 1 | NA | NA | NA | 1 | 1 | 1 |
| **FSYL** | *Fagus sylvatica* | 1 | 1 | 1 | 1 | 1 | 1 | 1 | 1 |
| **GETN** | *Genista etnensis* | 1 | 1 | 1 | NA | NA | 1 | 1 | 1 |
| **GTYR** | *Genista tyrrhena* | 1 | 1 | 1 | NA | NA | NA | 1 | 1 |
| **IAQU** | *Ilex aquifolium* | 1 | 1 | 1 | 1 | 1 | 1 | 1 | 1 |
| **JCOM** | *Juniperus communis* | 1 | 1 | 1 | 1 | 1 | 1 | 1 | 1 |
| **JDEL** | *Juniperus deltoides* ( *J. oxycedrus aggr.)* | 1 | 1 | 1 | NA | NA | 1 | NA | 1 |
| **JDRU** | *Juniperus drupacea* | 1 | 1 | NA | NA | NA | 1 | 1 | 1 |
| **JEXC** | *Juniperus excelsa* | 1 | 1 | 1 | NA | 1 | 1 | 1 | 1 |
| **JFOE** | *Juniperus foetidissima* | 1 | 1 | 1 | NA | 1 | 1 | 1 | 1 |
| **JMAC** | *Juniperus macrocarpa* | 1 | 1 | 1 | NA | NA | 1 | 1 | 1 |
| **JNAV** | *Juniperus navicularis* | 1 | 1 | 1 | 1 | NA | 1 | 1 | 1 |
| **JOXY** | ***Juniperus oxycedrus*** | 1 | 1 | 1 | 1 | 1 | 1 | 1 | 1 |
| **JPHO** | *Juniperus phoenicea* | 1 | 1 | 1 | 1 | 1 | 1 | 1 | 1 |
| **JREG** | *Juglans regia* | 1 | 1 | 1 | 1 | 1 | 1 | 1 | 1 |
| **JTHU** | *Juniperus thurifera* | 1 | 1 | 1 | NA | 1 | 1 | 1 | 1 |
| **LARB** | *Lonicera arborea* | 1 | 1 | 1 | NA | NA | 1 | 1 | 1 |
| **LNOB** | *Laurus nobilis* | 1 | 1 | 1 | 1 | 1 | 1 | 1 | 1 |
| **LORI** | *Liquidambar orientalis* | 1 | 1 | 1 | NA | NA | 1 | 1 | 1 |
| **MCOM** | *Myrtus communis* | 1 | 1 | 1 | 1 | 1 | 1 | 1 | 1 |
| **MDAS** | *Malus dasyphylla* | 1 | NA | NA | NA | NA | 1 | 1 | 1 |
| **MFAY** | *Myrica faya* | 1 | 1 | NA | NA | 1 | 1 | 1 | 1 |
| **MFLO** | *Malus florentina* | 1 | 1 | NA | NA | NA | 1 | 1 | 1 |
| **MSYL** | *Malus sylvestris* | 1 | 1 | 1 | 1 | 1 | 1 | 1 | 1 |
| **MTRI** | *Malus trilobata* | 1 | 1 | NA | NA | NA | 1 | 1 | 1 |
| **NOLE** | *Nerium oleander* | 1 | 1 | 1 | 1 | 1 | 1 | 1 | 1 |
| **OCAR** | *Ostrya carpinifolia* | 1 | 1 | 1 | 1 | 1 | 1 | 1 | 1 |
| **OEUR** | *Olea europaea* | 1 | 1 | 1 | 1 | 1 | 1 | 1 | 1 |
| **PALB** | *Populus alba* | 1 | 1 | 1 | 1 | 1 | 1 | 1 | 1 |
| **PANG** | *Phillyrea angustifolia* | 1 | 1 | 1 | 1 | 1 | 1 | 1 | 1 |
| **PATL** | *Pistacia atlantica* | 1 | 1 | 1 | NA | 1 | 1 | 1 | 1 |
| **PAVI** | *Prunus avium* | 1 | 1 | 1 | 1 | 1 | 1 | 1 | 1 |
| **PBOU** | *Pyrus bourgaeana* | 1 | 1 | 1 | 1 | NA | 1 | 1 | 1 |
| **PBRI** | *Prunus brigantina* | 1 | 1 | NA | NA | NA | 1 | 1 | 1 |
| **PBRU** | *Pinus brutia* | 1 | 1 | 1 | NA | 1 | 1 | 1 | 1 |
| **PCER** | *Prunus cerasifera* | 1 | 1 | 1 | 1 | 1 | 1 | 1 | 1 |
| **PCOC** | *Prunus cocomilia* | 1 | 1 | NA | NA | NA | 1 | 1 | 1 |
| **PCOR** | *Pyrus cordata* | 1 | 1 | 1 | NA | NA | 1 | 1 | 1 |
| **PELA** | *Pyrus elaeagrifolia* | 0 | 1 | 1 | NA | NA | 1 | 1 | 1 |
| **PHAL** | *Pinus halepensis* | 1 | 1 | 1 | 1 | 1 | 1 | 1 | 1 |
| **PHEL** | *Pinus heldreichii* | 1 | 1 | 1 | NA | NA | 1 | 1 | 1 |
| **PINI** | *Pinus nigra* | 1 | 1 | 1 | 1 | 1 | 1 | 1 | 1 |
| **PLAT** | *Phillyrea latifolia* | 1 | 1 | 1 | 1 | 1 | 1 | 1 | 1 |
| **PLEN** | *Pistacia lentiscus* | 1 | 1 | 1 | 1 | 1 | 1 | 1 | 1 |
| **PLUS** | *Prunus lusitanica* | 1 | 1 | 1 | NA | 1 | 1 | NA | 1 |
| **PMAH** | *Prunus mahaleb* | 1 | 1 | 1 | 1 | 1 | 1 | 1 | 1 |
| **PMUG** | ***Pinus mugo*** | 1 | 1 | 1 | 1 | NA | 1 | 1 | 1 |
| **PONI** | *Populus nigra* | 1 | 1 | 1 | 1 | 1 | 1 | 1 | 1 |
| **PORI** | *Platanus orientalis* | 1 | 1 | 1 | NA | 1 | 1 | 1 | 1 |
| **PPAD** | *Prunus padus* | 1 | 1 | 1 | 1 | 1 | 1 | 1 | 1 |
| **PPIA** | *Pinus pinea* | 1 | 1 | 1 | 1 | 1 | 1 | 1 | 1 |
| **PPIR** | *Pinus pinaster* | 1 | 1 | 1 | 1 | 1 | 1 | 1 | 1 |
| **PPYR** | *Pyrus pyraster* | 1 | 1 | 1 | NA | NA | 1 | NA | 1 |
| **PSPI** | *Pyrus spinosa* | 1 | 1 | 1 | NA | NA | 1 | 1 | 1 |
| **PSYL** | *Pinus sylvestris* | 1 | 1 | 1 | 1 | 1 | 1 | 1 | 1 |
| **PSYR** | *Pyrus syriaca* | 0 | 1 | 1 | NA | 1 | 1 | 1 | 1 |
| **PTER** | *Pistacia terebinthus* | 1 | 1 | 1 | 1 | 1 | NA | 1 | 1 |
| **PTHE** | *Phoenix theophrasti* | 1 | 1 | 1 | NA | NA | 1 | NA | 1 |
| **PTRE** | *Populus tremula* | 1 | 1 | 1 | 1 | 1 | 1 | 1 | 1 |
| **PUNC** | *Pinus uncinata* (*P mugo* aggr.) | 1 | 1 | 1 | 1 | 1 | 1 | 1 | 1 |
| **PWEB** | *Prunus webbii* | 1 | 1 | 1 | NA | NA | 1 | 1 | 1 |
| **QALN** | *Quercus alnifolia* | 1 | 1 | 1 | NA | 1 | 1 | 1 | 1 |
| **QAUC** | *Quercus aucheri* | 1 | 1 | NA | NA | NA | 1 | 1 | 1 |
| **QCAN** | *Quercus canariensis* | 1 | 1 | 1 | 1 | NA | 1 | 1 | 1 |
| **QCER** | *Quercus cerris* | 1 | 1 | 1 | 1 | 1 | 1 | 1 | 1 |
| **QCOC** | *Quercus coccifera* | 1 | 1 | 1 | 1 | 1 | 1 | 1 | 1 |
| **QCON** | *Quercus congesta* | 1 | 1 | NA | NA | NA | 1 | 1 | NA |
| **QCRE** | *Quercus crenata* | 1 | 1 | NA | NA | NA | 1 | 1 | 1 |
| **QDAL** | *Quercus dalechampii* | 1 | 1 | NA | NA | NA | 1 | 1 | 1 |
| **QFAG** | *Quercus faginea* | 1 | 1 | 1 | 1 | 1 | 1 | 1 | 1 |
| **QFRA** | *Quercus frainetto* | 1 | 1 | 1 | 1 | NA | 1 | 1 | 1 |
| **QGUS** | *Quercus gussonei* | 1 | 1 | NA | NA | NA | 1 | 1 | 1 |
| **QICH** | *Quercus ichnusae* | 1 | 1 | NA | NA | NA | 1 | 1 | 1 |
| **QILE** | *Quercus ilex* | 1 | 1 | 1 | 1 | 1 | 1 | 1 | 1 |
| **QINF** | *Quercus infectoria* | 1 | NA | NA | NA | NA | 1 | 1 | 1 |
| **QITH** | *Quercus ithaburensis* | 1 | 1 | NA | NA | NA | 1 | 1 | 1 |
| **QPET** | *Quercus petraea* | 1 | 1 | 1 | 1 | 1 | 1 | 1 | 1 |
| **QPUB** | *Quercus pubescens* | 1 | 1 | 1 | 1 | 1 | 1 | 1 | 1 |
| **QPYR** | *Quercus pyrenaica* | 1 | 1 | 1 | 1 | 1 | 1 | 1 | NA |
| **QROB** | *Quercus robur* | 1 | 1 | 1 | 1 | 1 | 1 | 1 | 1 |
| **QSUB** | *Quercus suber* | 1 | 1 | 1 | 1 | 1 | 1 | 1 | 1 |
| **QTRO** | *Quercus trojana* | 1 | 1 | 1 | 1 | NA | 1 | 1 | 1 |
| **RALA** | *Rhamnus alaternus* | 1 | 1 | 1 | 1 | 1 | 1 | 1 | NA |
| **RCAT** | *Rhamnus cathartica* | 1 | 1 | 1 | 1 | 1 | 1 | 1 | 1 |
| **RPER** | *Rhamnus persicifolia* | 1 | 1 | 1 | NA | NA | 1 | 1 | 1 |
| **RPON** | *Rhododendron ponticum* | 1 | 1 | 1 | 1 | NA | 1 | 1 | 1 |
| **SALB** | *Salix alba* | 1 | 1 | 1 | 1 | 1 | 1 | 1 | 1 |
| **SAMP** | *Salix amplexicaulis* | 1 | 1 | NA | NA | NA | 1 | 1 | 1 |
| **SAPE** | *Salix apennina* | 1 | 1 | NA | NA | NA | 1 | 1 | 1 |
| **SAPP** | *Salix appendiculata* | 1 | 1 | 1 | NA | NA | NA | NA | NA |
| **SARI** | *Sorbus aria* | 1 | 1 | 1 | 1 | 1 | 1 | 1 | 1 |
| **SARR** | *Salix arrigonii* | 1 | 1 | NA | NA | NA | 1 | 1 | NA |
| **SATR** | *Salix triandra* | 1 | 1 | 1 | NA | NA | 1 | 1 | 1 |
| **SAUC** | *Sorbus aucuparia* | 1 | 1 | 1 | 1 | 1 | 1 | 1 | 1 |
| **SAUS** | *Sorbus austriaca* | 1 | 1 | NA | NA | NA | 1 | 1 | 1 |
| **SCAP** | *Salix caprea* | 1 | 1 | 1 | 1 | 1 | 1 | 1 | 1 |
| **SCIN** | *Salix cinerea* | 1 | 1 | 1 | NA | 1 | 1 | 1 | 1 |
| **SDOM** | *Sorbus domestica* | 1 | 1 | 1 | 1 | 1 | 1 | 1 | 1 |
| **SELE** | *Salix eleagnos* | 1 | 1 | 1 | NA | NA | 1 | NA | 1 |
| **SETR** | *Searsia tripartita* | 1 | 1 | NA | NA | NA | 1 | 1 | 1 |
| **SFRA** | *Salix fragilis* | 1 | 1 | 1 | 1 | 1 | 1 | 1 | 1 |
| **SGRA** | *Sorbus graeca* | 1 | NA | 1 | NA | 1 | 1 | 1 | 1 |
| **SGUS** | *Salix gussonei* | 1 | 1 | NA | NA | NA | 1 | 1 | NA |
| **SJUN** | *Spartium junceum* | 1 | 1 | 1 | NA | 1 | 1 | 1 | 1 |
| **SLAT** | *Sorbus latifolia* | 1 | 1 | 1 | NA | NA | 1 | 1 | 1 |
| **SMOU** | *Sorbus mougeotii* | 1 | 1 | 1 | NA | NA | 1 | 1 | 1 |
| **SNIG** | *Sambucus nigra* | 1 | 1 | 1 | 1 | 1 | 1 | 1 | 1 |
| **SOFF** | *Styrax officinalis* | 1 | 1 | 1 | NA | 1 | 1 | 1 | 1 |
| **SPED** | *Salix pedicellata* | 1 | 1 | NA | NA | NA | 1 | NA | 1 |
| **SPEN** | *Salix pentandra* | 1 | 1 | 1 | 1 | NA | 1 | 1 | 1 |
| **SPIN** | *Staphylea pinnata* | 1 | 1 | 1 | NA | NA | 1 | 1 | 1 |
| **SPUR** | *Salix purpurea* | 1 | 1 | 1 | 1 | 1 | 1 | 1 | 1 |
| **SRAC** | *Sambucus racemosa* | 1 | 1 | 1 | NA | NA | 1 | 1 | 1 |
| **SSAL** | *Salix salviifolia* | 1 | 1 | NA | NA | NA | 1 | 1 | 1 |
| **STOR** | *Sorbus torminalis* | 1 | 1 | 1 | 1 | 1 | 1 | 1 | 1 |
| **SUMB** | *Sorbus umbellata* | 1 | NA | 1 | NA | NA | 1 | 1 | 1 |
| **SVIM** | *Salix viminalis* | 1 | 1 | NA | 1 | NA | 1 | 1 | 1 |
| **SXAN** | *Salix xanthicola* | 1 | 1 | NA | NA | NA | 1 | 1 | 1 |
| **TAFR** | *Tamarix africana* | 1 | 1 | 1 | NA | NA | 1 | 1 | 1 |
| **TARB** | *Tamarix arborea* | 1 | NA | NA | NA | NA | 1 | 1 | 1 |
| **TART** | *Tetraclinis articulata* | 1 | 1 | 1 | 1 | 1 | 1 | 1 | 1 |
| **TBAC** | *Taxus baccata* | 1 | 1 | 1 | 1 | 1 | 1 | 1 | 1 |
| **TBOV** | *Tamarix boveana* | 1 | 1 | NA | NA | NA | 1 | 1 | 1 |
| **TCAN** | *Tamarix canariensis* | 1 | NA | NA | 1 | NA | NA | 1 | NA |
| **TCOR** | *Tilia cordata* | 1 | 1 | 1 | 1 | 1 | 1 | 1 | 1 |
| **TDAL** | *Tamarix dalmatica* | 1 | 1 | NA | NA | 1 | NA | NA | 1 |
| **TGAL** | *Tamarix gallica* | 1 | 1 | 1 | NA | NA | 1 | 1 | 1 |
| **THAM** | *Tamarix hampeana* | 1 | 1 | NA | NA | NA | NA | NA | NA |
| **TLAX** | *Tamarix laxa* | 1 | 1 | NA | NA | NA | 1 | 1 | 1 |
| **TMAS** | *Tamarix mascatensis* | 1 | 1 | NA | NA | NA | 1 | 1 | 1 |
| **TMIN** | *Tamarix minoa* | 1 | 1 | NA | NA | NA | NA | NA | NA |
| **TPAR** | *Tamarix parviflora* | 1 | 1 | NA | NA | NA | 1 | 1 | 1 |
| **TPAS** | *Tamarix passerinoides* | 0 | NA | NA | NA | NA | 1 | 1 | 1 |
| **TPLA** | *Tilia platyphyllos* | 1 | 1 | 1 | 1 | 1 | 1 | 1 | 1 |
| **TRAM** | *Tamarix ramosissima* | 1 | 1 | 1 | NA | NA | 1 | 1 | 1 |
| **TSMY** | *Tamarix smyrnensis* | 1 | 1 | NA | NA | 1 | 1 | 1 | 1 |
| **TTEG** | *Tamarix tetragyna* | 1 | 1 | NA | NA | 1 | 1 | 1 | 1 |
| **TTEN** | *Tamarix tetrandra* | 1 | 1 | 1 | NA | 1 | 1 | 1 | 1 |
| **TTOM** | *Tilia tomentosa* | 1 | 1 | 1 | NA | NA | 1 | NA | 1 |
| **UCAN** | *Ulmus canescens* | 1 | 1 | NA | NA | 1 | 1 | 1 | NA |
| **UGLA** | *Ulmus glabra* | 1 | 1 | 1 | 1 | 1 | 1 | 1 | 1 |
| **ULAE** | *Ulmus laevis* | 1 | 1 | 1 | 1 | 1 | 1 | 1 | 1 |
| **UMIN** | *Ulmus minor* | 1 | 1 | 1 | 1 | 1 | 1 | 1 | 1 |
| **UPRO** | *Ulmus procera* | 1 | 1 | NA | NA | 1 | 1 | 1 | 1 |
| **VAGN** | *Vitex agnus-castus* | 1 | 1 | 1 | 1 | 1 | 1 | 1 | 1 |
| **ZABE** | *Zelkova abelicea* | 1 | 1 | NA | NA | NA | 1 | 1 | 1 |
| **ZSIC** | *Zelkova sicula* | 1 | 1 | NA | NA | NA | 1 | 1 | 1 |
|  | **TOTAL number of species with available data** | **207 (203 when aggregated)** | **201** | **159** | **102** | **114** | **199** | **195** | **195** |
|  |  |  |  |  |  |  |  |  |  |
|  |  |  | **Total number of species with at least 1 functional trait data: 204** | | | | **Total number of species with at least 1 DNA-region sequence data: 204** | | |

***Supplementary Table 3: Summary of the occurrences (observed or modelled) provided by the WOODIV database and their quality.*** *Obs occ: indicates if the WOODIV database provides observed (1) occurrences or not (0); Mod occ: indicates if the WOODIV database provides modelled (1) occurrences or not (0); TSS and AUC: mean +/- SD of TSS and AUC among the 100 replicates for each modelled species; SQm: expert-based information (for modelling purpose) on the representativeness of the observed occurrences in Italy, Sardinia and Sicily (0: sufficient and 1: subsampled); SQ small-range sp.: expert-based information on the sampling quality (SQ) for small-ranged species (1: good). NA = Not Applicable.*

| **Species** | **Obs occ** | **Mod occ** | **TSS** | **AUC** | ***SQm Italy*** | ***SQm Sardinia*** | ***SQm Sicily*** | **SQ small-range sp.** |
| --- | --- | --- | --- | --- | --- | --- | --- | --- |
| *Abies alba* | 1 | 1 | 0.8 +/- 0.03 | 0.95 +/- 0.01 | 0 | NA | NA | NA |
| *Abies borisii-regis* | 1 | 1 | 0.82 +/- 0.06 | 0.94 +/- 0.03 | NA | NA | NA | NA |
| *Abies cephalonica* | 1 | 1 | 0.72 +/- 0.08 | 0.91 +/- 0.03 | NA | NA | NA | NA |
| *Abies nebrodensis* | 1 | 0 | NA | NA | NA | NA | NA | 1 |
| *Abies pinsapo* | 1 | 1 | 0.96 +/- 0.07 | 0.99 +/- 0.03 | NA | NA | NA | NA |
| *Acer campestre* | 1 | 1 | 0.7 +/- 0.03 | 0.9 +/- 0.01 | 0 | 1 | 0 | NA |
| *Acer cappadocicum* | 1 | 0 | NA | NA | 0 | NA | NA | 0 |
| *Acer heldreichii* | 1 | 1 | 0.88 +/- 0.08 | 0.97 +/- 0.03 | NA | NA | NA | NA |
| *Acer hyrcanum* | 1 | 1 | 0.76 +/- 0.06 | 0.92 +/- 0.04 | NA | NA | NA | NA |
| *Acer monspessulanum* | 1 | 1 | 0.45 +/- 0.03 | 0.78 +/- 0.01 | 1 | 1 | 1 | NA |
| *Acer obtusifolium* | 1 | 0 | NA | NA | NA | NA | NA | 1 |
| *Acer opalus* | 1 | 1 | 0.68 +/- 0.04 | 0.9 +/- 0.02 | 0 | NA | 0 | NA |
| *Acer platanoides* | 1 | 1 | 0.73 +/- 0.04 | 0.92 +/- 0.02 | 0 | NA | 0 | NA |
| *Acer pseudoplatanus* | 1 | 1 | 0.72 +/- 0.03 | 0.92 +/- 0.01 | 0 | NA | 0 | NA |
| *Acer sempervirens* | 1 | 1 | 0.75 +/- 0.08 | 0.92 +/- 0.03 | NA | NA | NA | NA |
| *Acer tataricum* | 1 | 1 | 0.9 +/- 0.04 | 0.97 +/- 0.02 | NA | NA | NA | NA |
| *Aesculus hippocastanum* | 1 | 1 | 0.81 +/- 0.08 | 0.95 +/- 0.03 | NA | NA | NA | NA |
| *Alnus cordata* | 1 | 1 | 0.96 +/- 0.06 | 0.99 +/- 0.02 | 0 | 1 | NA | NA |
| *Alnus glutinosa* aggr. | 1 | 1 | 0.7 +/- 0.03 | 0.91 +/- 0.01 | 0 | 1 | 0 | NA |
| *Alnus incana* | 1 | 1 | 0.82 +/- 0.05 | 0.96 +/- 0.02 | 0 | NA | NA | NA |
| *Alnus orientalis* | 1 | 0 | NA | NA | NA | NA | NA | 1 |
| *Alnus viridis* | 1 | 1 | 0.77 +/- 0.11 | 0.91 +/- 0.06 | 1 | NA | NA | NA |
| *Arbutus andrachne* | 1 | 1 | 0.61 +/- 0.07 | 0.86 +/- 0.03 | NA | NA | NA | NA |
| *Arbutus unedo* | 1 | 1 | 0.42 +/- 0.02 | 0.77 +/- 0.01 | 1 | 1 | 0 | NA |
| *Betula pendula* | 1 | 1 | 0.65 +/- 0.05 | 0.89 +/- 0.02 | 1 | NA | 0 | NA |
| *Betula pubescens* | 1 | 1 | 0.66 +/- 0.07 | 0.88 +/- 0.03 | 0 | NA | NA | NA |
| *Buxus balearica* | 1 | 1 | 0.81 +/- 0.14 | 0.93 +/- 0.07 | NA | NA | NA | NA |
| *Buxus sempervirens* | 1 | 1 | 0.64 +/- 0.03 | 0.88 +/- 0.01 | 0 | NA | NA | NA |
| *Carpinus betulus* | 1 | 1 | 0.75 +/- 0.05 | 0.92 +/- 0.03 | 0 | NA | NA | NA |
| *Carpinus orientalis* | 1 | 1 | 0.68 +/- 0.03 | 0.91 +/- 0.01 | 1 | NA | NA | NA |
| *Castanea sativa* | 1 | 1 | 0.68 +/- 0.04 | 0.9 +/- 0.02 | NA | NA | NA | NA |
| *Cedrus libani* | 1 | 0 | NA | NA | NA | NA | NA | 1 |
| *Celtis australis* | 1 | 1 | 0.41 +/- 0.03 | 0.77 +/- 0.01 | 1 | 1 | 1 | NA |
| *Celtis planchoniana* | 1 | 0 | NA | NA | NA | NA | NA | 0 |
| *Celtis tournefortii* | 1 | 1 | 0.58 +/- 0.15 | 0.8 +/- 0.09 | NA | NA | NA | NA |
| *Ceratonia siliqua* | 1 | 1 | 0.67 +/- 0.04 | 0.9 +/- 0.02 | 1 | 1 | 0 | NA |
| *Cercis siliquastrum* | 1 | 1 | 0.61 +/- 0.04 | 0.87 +/- 0.02 | 1 | NA | 0 | NA |
| *Chamaerops humilis* | 1 | 1 | 0.74 +/- 0.03 | 0.93 +/- 0.01 | 1 | 1 | 0 | NA |
| *Corylus avellana* | 1 | 1 | 0.47 +/- 0.02 | 0.8 +/- 0.01 | 1 | 1 | 1 | NA |
| *Corylus colurna* | 1 | 1 | 0.81 +/- 0.08 | 0.95 +/- 0.03 | NA | NA | NA | NA |
| *Cotinus coggygria* | 1 | 1 | 0.64 +/- 0.05 | 0.89 +/- 0.02 | 1 | NA | NA | NA |
| *Cotoneaster granatensis* | 1 | 1 | 0.85 +/- 0.08 | 0.96 +/- 0.03 | NA | NA | NA | NA |
| *Crataegus azarolus* | 1 | 1 | 0.97 +/- 0.07 | 0.99 +/- 0.03 | NA | NA | NA | NA |
| *Crataegus heldreichii* | 1 | 1 | 0.73 +/- 0.07 | 0.92 +/- 0.03 | NA | NA | NA | NA |
| *Crataegus laciniata* | 1 | 1 | 0.84 +/- 0.1 | 0.93 +/- 0.05 | NA | NA | NA | NA |
| *Crataegus laevigata* | 1 | 1 | 0.67 +/- 0.06 | 0.89 +/- 0.03 | 1 | NA | 1 | NA |
| *Crataegus monogyna* | 1 | 1 | 0.25 +/- 0.02 | 0.67 +/- 0.01 | 1 | 1 | 0 | NA |
| *Crataegus nevadensis* | 1 | 1 | 0.77 +/- 0.11 | 0.92 +/- 0.05 | NA | NA | NA | NA |
| *Crataegus orientalis* | 1 | 1 | 0.68 +/- 0.09 | 0.89 +/- 0.04 | NA | NA | NA | NA |
| *Crataegus pentagyna* | 1 | 0 | NA | NA | NA | NA | NA | 0 |
| *Crataegus pycnoloba* | 1 | 0 | NA | NA | NA | NA | NA | 1 |
| *Cupressus sempervirens* | 1 | 1 | 0.67 +/- 0.06 | 0.89 +/- 0.03 | NA | NA | NA | NA |
| *Cytisus aeolicus* | 1 | 0 | NA | NA | NA | NA | NA | 1 |
| *Erica arborea* | 1 | 1 | 0.44 +/- 0.02 | 0.78 +/- 0.01 | 1 | 1 | 0 | NA |
| *Fagus orientalis* | 1 | 1 | 0.86 +/- 0.12 | 0.95 +/- 0.06 | NA | NA | NA | NA |
| *Fagus sylvatica* | 1 | 1 | 0.69 +/- 0.03 | 0.9 +/- 0.01 | 0 | NA | NA | NA |
| *Ficus carica* | 1 | 1 | 0.35 +/- 0.03 | 0.72 +/- 0.01 | 1 | 1 | 0 | NA |
| *Fontanesia philly~~i~~raeoides* | 1 | 0 | NA | NA | NA | NA | 0 | 1 |
| *Frangula alnus* | 1 | 1 | 0.51 +/- 0.03 | 0.82 +/- 0.02 | 0 | NA | NA | NA |
| *Fraxinus angustifolia* | 1 | 1 | 0.37 +/- 0.02 | 0.74 +/- 0.01 | 1 | 1 | 0 | NA |
| *Fraxinus excelsior* | 1 | 1 | 0.67 +/- 0.03 | 0.89 +/- 0.01 | 1 | NA | 0 | NA |
| *Fraxinus ornus* | 1 | 1 | 0.64 +/- 0.03 | 0.88 +/- 0.01 | 0 | 1 | 0 | NA |
| *Fraxinus pallisae* | 1 | 0 | NA | NA | NA | NA | NA | 1 |
| *Genista etnensis* | 1 | 0 | NA | NA | NA | NA | NA | 0 |
| *Genista tyrrhena* | 1 | 0 | NA | NA | 0 | NA | 0 | 1 |
| *Ilex aquifolium* | 1 | 1 | 0.54 +/- 0.03 | 0.83 +/- 0.02 | 1 | 1 | 0 | NA |
| *Juglans regia* | 1 | 1 | 0.97 +/- 0.04 | 0.99 +/- 0.01 | NA | NA | NA | NA |
| *Juniperus communis* | 1 | 1 | 0.6 +/- 0.03 | 0.86 +/- 0.01 | 1 | 1 | NA | NA |
| *Juniperus drupacea* | 1 | 1 | 0.82 +/- 0.19 | 0.91 +/- 0.13 | NA | NA | NA | NA |
| *Juniperus excelsa* | 1 | 1 | 0.83 +/- 0.12 | 0.92 +/- 0.06 | 1 | NA | NA | NA |
| *Juniperus foetidissima* | 1 | 1 | 0.7 +/- 0.08 | 0.9 +/- 0.03 | NA | NA | NA | NA |
| *Juniperus macrocarpa* | 1 | 1 | 0.79 +/- 0.06 | 0.94 +/- 0.03 | 1 | 1 | 1 | NA |
| *Juniperus navicularis* | 1 | 0 | NA | NA | NA | NA | NA | 0 |
| *Juniperus oxycedrus aggr.* | 1 | 1 | 0.37 +/- 0.02 | 0.74 +/- 0.01 | 1 | 1 | NA | NA |
| *Juniperus phoenicea* | 1 | 1 | 0.5 +/- 0.02 | 0.81 +/- 0.01 | 1 | 1 | 0 | NA |
| *Juniperus thurifera* | 1 | 1 | 0.75 +/- 0.03 | 0.93 +/- 0.01 | 1 | NA | NA | NA |
| *Laurus nobilis* | 1 | 1 | 0.51 +/- 0.03 | 0.82 +/- 0.02 | 1 | 0 | 0 | NA |
| *Liquidambar orientalis* | 1 | 0 | NA | NA | NA | NA | NA | 1 |
| *Lonicera arborea* | 1 | 1 | 0.88 +/- 0.07 | 0.97 +/- 0.03 | NA | NA | NA | NA |
| *Malus dasyphylla* | 1 | 1 | 0.86 +/- 0.07 | 0.96 +/- 0.03 | NA | NA | NA | NA |
| *Malus florentina* | 1 | 1 | 0.84 +/- 0.06 | 0.94 +/- 0.03 | 0 | NA | NA | NA |
| *Malus sylvestris* | 1 | 1 | 0.58 +/- 0.03 | 0.86 +/- 0.02 | 1 | 1 | 0 | NA |
| *Malus trilobata* | 1 | 0 | NA | NA | NA | NA | NA | 1 |
| *Myrica faya* | 1 | 1 | 0.99 +/- 0.04 | 1 +/- 0 | NA | NA | NA | NA |
| *Myrtus communis* | 1 | 1 | 0.56 +/- 0.03 | 0.84 +/- 0.01 | 1 | 1 | 0 | NA |
| *Nerium oleander* | 1 | 1 | 0.61 +/- 0.03 | 0.86 +/- 0.01 | 1 | 1 | 0 | NA |
| *Olea europaea* | 1 | 1 | 0.47 +/- 0.02 | 0.79 +/- 0.01 | 1 | 1 | 0 | NA |
| *Ostrya carpinifolia* | 1 | 1 | 0.7 +/- 0.04 | 0.9 +/- 0.02 | 0 | 1 | 0 | NA |
| *Phillyrea angustifolia* | 1 | 1 | 0.48 +/- 0.02 | 0.8 +/- 0.01 | 1 | 1 | NA | NA |
| *Phillyrea latifolia* | 1 | 1 | 0.41 +/- 0.02 | 0.76 +/- 0.01 | 1 | 1 | 0 | NA |
| *Phoenix theophrasti* | 1 | 1 | 0.97 +/- 0.09 | 0.98 +/- 0.06 | NA | NA | NA | NA |
| *Pinus brutia* | 1 | 1 | 0.73 +/- 0.08 | 0.92 +/- 0.03 | NA | NA | NA | NA |
| *Pinus halepensis* | 1 | 1 | 0.55 +/- 0.02 | 0.84 +/- 0.01 | 1 | 1 | 0 | NA |
| *Pinus heldreichii* | 1 | 1 | 0.87 +/- 0.08 | 0.96 +/- 0.03 | 0 | NA | NA | NA |
| *Pinus mugo agg.* | 1 | 1 | 0.83 +/- 0.05 | 0.96 +/- 0.02 | 0 | NA | NA | NA |
| *Pinus nigra* | 1 | 1 | 0.51 +/- 0.04 | 0.82 +/- 0.02 | 0 | NA | 0 | NA |
| *Pinus pinaster* | 1 | 1 | 0.48 +/- 0.02 | 0.8 +/- 0.01 | 0 | 0 | 0 | NA |
| *Pinus pinea* | 1 | 1 | 0.49 +/- 0.02 | 0.81 +/- 0.01 | 1 | 1 | 0 | NA |
| *Pinus sylvestris* | 1 | 1 | 0.71 +/- 0.03 | 0.91 +/- 0.01 | 0 | NA | NA | NA |
| *Pistacia atlantica* | 1 | 0 | NA | NA | NA | NA | NA | 1 |
| *Pistacia lentiscus* | 1 | 1 | 0.52 +/- 0.02 | 0.82 +/- 0.01 | 1 | 1 | 0 | NA |
| *Pistacia terebinthus* | 1 | 1 | 0.33 +/- 0.02 | 0.72 +/- 0.01 | 1 | 1 | 0 | NA |
| *Platanus orientalis* | 1 | 1 | 0.68 +/- 0.04 | 0.89 +/- 0.02 | 1 | NA | 0 | NA |
| *Populus alba* | 1 | 1 | 0.36 +/- 0.02 | 0.73 +/- 0.01 | 1 | 1 | 0 | NA |
| *Populus nigra* | 1 | 1 | 0.55 +/- 0.03 | 0.84 +/- 0.02 | 1 | 1 | 0 | NA |
| *Populus tremula* | 1 | 1 | 0.53 +/- 0.03 | 0.84 +/- 0.02 | 1 | 1 | 0 | NA |
| *Prunus avium* | 1 | 1 | 0.56 +/- 0.03 | 0.84 +/- 0.01 | 0 | 1 | NA | NA |
| *Prunus brigantina* | 1 | 1 | 0.96 +/- 0.06 | 0.99 +/- 0.02 | 0 | NA | NA | NA |
| *Prunus cerasifera* | 1 | 1 | 0.79 +/- 0.06 | 0.94 +/- 0.02 | NA | NA | NA | NA |
| *Prunus cocomilia* | 1 | 1 | 0.73 +/- 0.06 | 0.92 +/- 0.03 | 1 | NA | 0 | NA |
| *Prunus lusitanica* | 1 | 1 | 0.72 +/- 0.1 | 0.9 +/- 0.05 | NA | NA | NA | NA |
| *Prunus mahaleb* | 1 | 1 | 0.55 +/- 0.03 | 0.84 +/- 0.02 | 1 | NA | 0 | NA |
| *Prunus padus* | 1 | 1 | 0.73 +/- 0.11 | 0.89 +/- 0.06 | 0 | NA | NA | NA |
| *Prunus webbii* | 1 | 1 | 0.7 +/- 0.05 | 0.91 +/- 0.02 | 1 | NA | 0 | NA |
| *Pyrus bourgaeana* | 1 | 1 | 0.65 +/- 0.04 | 0.88 +/- 0.02 | NA | NA | NA | NA |
| *Pyrus cordata* | 1 | 1 | 0.8 +/- 0.1 | 0.92 +/- 0.05 | NA | NA | NA | NA |
| *Pyrus elaeagrifolia* | 0 | 0 | NA | NA | NA | NA | NA | 0 |
| *Pyrus pyraster* | 1 | 1 | 0.65 +/- 0.04 | 0.89 +/- 0.02 | 1 | 1 | 0 | NA |
| *Pyrus spinosa* | 1 | 1 | 0.64 +/- 0.03 | 0.88 +/- 0.01 | 0 | 0 | 0 | NA |
| *Pyrus syriaca* | 0 | 0 | NA | NA | NA | NA | NA | 0 |
| *Quercus alnifolia* | 1 | 1 | 0.92 +/- 0.11 | 0.97 +/- 0.05 | NA | NA | NA | NA |
| *Quercus aucheri* | 1 | 1 | 0.96 +/- 0.11 | 0.97 +/- 0.08 | NA | NA | NA | NA |
| *Quercus canariensis* | 1 | 1 | 0.8 +/- 0.08 | 0.94 +/- 0.03 | NA | NA | NA | NA |
| *Quercus cerris* | 1 | 1 | 0.76 +/- 0.05 | 0.93 +/- 0.02 | 0 | NA | 0 | NA |
| *Quercus coccifera* | 1 | 1 | 0.45 +/- 0.02 | 0.78 +/- 0.01 | 1 | 1 | 1 | NA |
| *Quercus congesta* | 1 | 1 | 0.86 +/- 0.08 | 0.96 +/- 0.03 | 1 | 1 | 1 | NA |
| *Quercus crenata* | 1 | 1 | 0.69 +/- 0.13 | 0.87 +/- 0.07 | 1 | NA | NA | NA |
| *Quercus dalechampii* | 1 | 1 | 0.64 +/- 0.06 | 0.89 +/- 0.03 | 1 | 1 | 0 | NA |
| *Quercus faginea* | 1 | 1 | 0.43 +/- 0.03 | 0.78 +/- 0.02 | NA | NA | NA | NA |
| *Quercus frainetto* | 1 | 1 | 0.76 +/- 0.05 | 0.93 +/- 0.02 | 0 | NA | NA | NA |
| *Quercus gussonei* | 1 | 0 | NA | NA | NA | NA | NA | 1 |
| *Quercus ichnusae* | 1 | 0 | NA | NA | NA | NA | NA | 1 |
| *Quercus ilex* | 1 | 1 | 0.33 +/- 0.02 | 0.7 +/- 0.01 | 0 | 0 | 0 | NA |
| *Quercus infectoria* | 1 | 1 | 0.8 +/- 0.2 | 0.91 +/- 0.11 | NA | NA | NA | NA |
| *Quercus ithaburensis* | 1 | 1 | 0.68 +/- 0.06 | 0.91 +/- 0.03 | 1 | NA | NA | NA |
| *Quercus petraea* | 1 | 1 | 0.65 +/- 0.04 | 0.88 +/- 0.02 | 0 | NA | 0 | NA |
| *Quercus pubescens* | 1 | 1 | 0.63 +/- 0.02 | 0.87 +/- 0.01 | 0 | 0 | 0 | NA |
| *Quercus pyrenaica* | 1 | 1 | 0.68 +/- 0.03 | 0.9 +/- 0.01 | 1 | NA | NA | NA |
| *Quercus robur* | 1 | 1 | 0.62 +/- 0.04 | 0.87 +/- 0.02 | 0 | NA | NA | NA |
| *Quercus suber* | 1 | 1 | 0.6 +/- 0.02 | 0.86 +/- 0.01 | 1 | 0 | 0 | NA |
| *Quercus trojana* | 1 | 1 | 0.84 +/- 0.06 | 0.95 +/- 0.02 | 0 | NA | NA | NA |
| *Rhamnus alaternus* | 1 | 1 | 0.43 +/- 0.02 | 0.78 +/- 0.01 | 0 | 0 | 1 | NA |
| *Rhamnus cathartica* | 1 | 1 | 0.65 +/- 0.04 | 0.89 +/- 0.02 | 1 | NA | 1 | NA |
| *Rhamnus persicifolia* | 1 | 0 | NA | NA | NA | NA | NA | 1 |
| *Rhododendron ponticum* | 1 | 1 | 0.92 +/- 0.09 | 0.97 +/- 0.03 | NA | NA | NA | NA |
| *Salix alba* | 1 | 1 | 0.31 +/- 0.02 | 0.7 +/- 0.01 | 1 | 1 | 0 | NA |
| *Salix amplexicaulis* | 1 | 1 | 0.77 +/- 0.05 | 0.94 +/- 0.02 | NA | NA | NA | NA |
| *Salix apennina* | 1 | 0 | NA | NA | 0 | NA | 0 | 0 |
| *Salix appendiculata* | 1 | 1 | 0.8 +/- 0.12 | 0.91 +/- 0.06 | 1 | NA | NA | NA |
| *Salix arrigonii* | 1 | 0 | NA | NA | NA | NA | NA | 0 |
| *Salix caprea* | 1 | 1 | 0.65 +/- 0.03 | 0.88 +/- 0.01 | 0 | NA | 0 | NA |
| *Salix cinerea* | 1 | 1 | 0.62 +/- 0.04 | 0.87 +/- 0.02 | 1 | 1 | NA | NA |
| *Salix eleagnos* | 1 | 1 | 0.49 +/- 0.04 | 0.81 +/- 0.02 | 1 | NA | NA | NA |
| *Salix fragilis* | 1 | 1 | 0.37 +/- 0.03 | 0.74 +/- 0.02 | 1 | NA | NA | NA |
| *Salix gussonei* | 1 | 0 | NA | NA | NA | NA | NA | 1 |
| *Salix pedicellata* | 1 | 1 | 0.76 +/- 0.04 | 0.93 +/- 0.02 | 1 | 1 | 0 | NA |
| *Salix pentandra* | 1 | 1 | 0.51 +/- 0.23 | 0.72 +/- 0.15 | 1 | NA | NA | NA |
| *Salix purpurea* | 1 | 1 | 0.38 +/- 0.03 | 0.74 +/- 0.01 | 1 | 1 | 0 | NA |
| *Salix salviifolia* | 1 | 1 | 0.49 +/- 0.04 | 0.8 +/- 0.03 | NA | NA | NA | NA |
| *Salix triandra* | 1 | 1 | 0.38 +/- 0.04 | 0.75 +/- 0.02 | 1 | NA | NA | NA |
| *Salix viminalis* | 1 | 1 | 0.75 +/- 0.1 | 0.91 +/- 0.05 | NA | NA | NA | NA |
| *Salix xanthicola* | 1 | 0 | NA | NA | NA | NA | NA | 1 |
| *Sambucus nigra* | 1 | 1 | 0.38 +/- 0.02 | 0.75 +/- 0.01 | 1 | 1 | 0 | NA |
| *Sambucus racemosa* | 1 | 1 | 0.83 +/- 0.05 | 0.94 +/- 0.02 | 0 | NA | NA | NA |
| *Searsia tripartita* | 1 | 0 | NA | NA | NA | NA | NA | 1 |
| *Sorbus aria* | 1 | 1 | 0.62 +/- 0.03 | 0.88 +/- 0.01 | 0 | 1 | 1 | NA |
| *Sorbus aucuparia* | 1 | 1 | 0.65 +/- 0.03 | 0.89 +/- 0.01 | 1 | 1 | 0 | NA |
| *Sorbus austriaca* | 1 | 1 | 0.92 +/- 0.05 | 0.98 +/- 0.02 | NA | NA | NA | NA |
| *Sorbus domestica* | 1 | 1 | 0.52 +/- 0.03 | 0.82 +/- 0.02 | 1 | 1 | 0 | NA |
| *Sorbus graeca* | 1 | 1 | 0.75 +/- 0.06 | 0.93 +/- 0.03 | 1 | NA | 0 | NA |
| *Sorbus latifolia* | 1 | 1 | 0.71 +/- 0.13 | 0.9 +/- 0.06 | NA | NA | NA | NA |
| *Sorbus mougeotii* | 1 | 1 | 0.92 +/- 0.06 | 0.97 +/- 0.03 | 1 | NA | NA | NA |
| *Sorbus torminalis* | 1 | 1 | 0.53 +/- 0.03 | 0.82 +/- 0.01 | 1 | 0 | 1 | NA |
| *Sorbus umbellata* | 1 | 1 | 0.72 +/- 0.06 | 0.91 +/- 0.03 | NA | NA | NA | NA |
| *Spartium junceum* | 1 | 1 | 0.54 +/- 0.03 | 0.84 +/- 0.01 | 1 | 1 | 0 | NA |
| *Staphylea pinnata* | 1 | 1 | 0.98 +/- 0.06 | 0.99 +/- 0.02 | 0 | NA | NA | NA |
| *Styrax officinalis* | 1 | 1 | 0.73 +/- 0.08 | 0.92 +/- 0.03 | 1 | NA | NA | NA |
| *Tamarix africana* | 1 | 1 | 0.56 +/- 0.03 | 0.84 +/- 0.01 | 1 | 1 | 0 | NA |
| *Tamarix arborea* | 1 | 0 | NA | NA | NA | NA | NA | 0 |
| *Tamarix boveana* | 1 | 1 | 0.89 +/- 0.07 | 0.97 +/- 0.02 | NA | NA | NA | NA |
| *Tamarix canariensis* | 1 | 1 | 0.67 +/- 0.04 | 0.9 +/- 0.02 | 1 | 1 | 0 | NA |
| *Tamarix dalmatica* | 1 | 1 | 0.75 +/- 0.09 | 0.92 +/- 0.04 | 1 | 1 | NA | NA |
| *Tamarix gallica* | 1 | 1 | 0.5 +/- 0.04 | 0.83 +/- 0.02 | 1 | 1 | 0 | NA |
| *Tamarix hampeana* | 1 | 1 | 0.78 +/- 0.09 | 0.93 +/- 0.03 | NA | NA | NA | NA |
| *Tamarix laxa* | 1 | 0 | NA | NA | NA | NA | NA | 1 |
| *Tamarix mascatensis* | 1 | 0 | NA | NA | NA | NA | NA | 0 |
| *Tamarix minoa* | 1 | 0 | NA | NA | NA | NA | NA | 0 |
| *Tamarix parviflora* | 1 | 1 | 0.75 +/- 0.07 | 0.92 +/- 0.03 | NA | NA | NA | NA |
| *Tamarix passerinoides* | 0 | 0 | NA | NA | NA | NA | NA | 0 |
| *Tamarix ramosissima* | 1 | 1 | 0.69 +/- 0.25 | 0.84 +/- 0.15 | NA | NA | NA | NA |
| *Tamarix smyrnensis* | 1 | 1 | 0.67 +/- 0.15 | 0.87 +/- 0.09 | NA | NA | NA | NA |
| *Tamarix tetragyna* | 1 | 0 | NA | NA | NA | NA | NA | 0 |
| *Tamarix tetrandra* | 1 | 1 | 0.67 +/- 0.1 | 0.88 +/- 0.05 | NA | NA | NA | NA |
| *Taxus baccata* | 1 | 1 | 0.49 +/- 0.04 | 0.81 +/- 0.02 | 0 | 0 | 0 | NA |
| *Tetraclinis articulata* | 1 | 0 | NA | NA | NA | NA | NA | 1 |
| *Tilia cordata* | 1 | 1 | 0.7 +/- 0.05 | 0.91 +/- 0.02 | 1 | NA | NA | NA |
| *Tilia platyphyllos* | 1 | 1 | 0.68 +/- 0.04 | 0.9 +/- 0.02 | 1 | NA | 0 | NA |
| *Tilia tomentosa* | 1 | 1 | 0.82 +/- 0.07 | 0.94 +/- 0.03 | NA | NA | NA | NA |
| *Ulmus canescens* | 1 | 1 | 0.62 +/- 0.11 | 0.84 +/- 0.07 | 1 | 1 | 0 | NA |
| *Ulmus glabra* | 1 | 1 | 0.52 +/- 0.05 | 0.83 +/- 0.02 | 1 | NA | 0 | NA |
| *Ulmus laevis* | 1 | 1 | 0.8 +/- 0.06 | 0.95 +/- 0.02 | 1 | NA | NA | NA |
| *Ulmus minor* | 1 | 1 | 0.34 +/- 0.02 | 0.72 +/- 0.01 | 1 | 1 | 0 | NA |
| *Ulmus procera* | 1 | 1 | 0.72 +/- 0.11 | 0.88 +/- 0.06 | NA | NA | NA | NA |
| *Vitex agnus-castus* | 1 | 1 | 0.61 +/- 0.03 | 0.88 +/- 0.02 | 1 | 0 | 1 | NA |
| *Zelkova abelicea* | 1 | 1 | 0.96 +/- 0.1 | 0.97 +/- 0.07 | NA | NA | NA | NA |
| *Zelkova sicula* | 1 | 0 | NA | NA | NA | NA | NA | 1 |
|  | **203 sp.** | **171 sp.** |  |  |  |  |  |  |

***Supplementary Table 4: Sources, type and geographic origin of samples used for DNA region extraction and/or sequencing.*** *Sources and type of samples gathered by the WOODIV consortium to extract and/or sequence DNA regions which were unavailable in GenBank.*

| **Species** | **Type of sample** | **Source** |
| --- | --- | --- |
| *Abies alba* | dry leaves | France (B. Fady) |
| *Abies borisii-regis* | dry leaves | Greece (Common garden in France, B. Fady) |
| *Abies cephalonica* | dry leaves | Greece (Common garden in France, B. Fady) |
| *Abies nebrodensis* | Herbarium (DNA) | Italy (B. Fady) |
| *Abies pinsapo* | Herbarium (DNA) | Spain (B. Fady) |
| *Acer campestre* | dry leaves | France (B. Fady) |
| *Acer cappadocicum* | dry leaves | Turkey (Arboretum National des Barres, France) |
| *Acer heldreichii* | dry leaves | Greece (Arboretum National des Barres, France) |
| *Acer hyrcanum* | dry leaves | Turkey (Arboretum National des Barres, France) |
| *Acer monspessulanum* | dry leaves | France (B. Fady) |
| *Acer obtusifolium* | dry leaves | Cyprus (Arboretum National des Barres, France) |
| *Acer opalus* | dry leaves | France (B. Fady) |
| *Acer platanoides* | dry leaves | France (B. Fady) |
| *Acer sempervirens* | Herbarium (DNA) | Berlin Botanic Garden |
| *Aesculus hippocastanum* | dry leaves | France (B. Fady) |
| *Alnus cordata* | dry leaves | France (B. Fady) |
| *Alnus rohlenae* | dry leaves | Montenegro (F. Médail) |
| *Arbutus andrachne* | dry leaves | Cyprus (N. Eliades) |
| *Arbutus unedo* | dry leaves | France (B. Fady) |
| *Betula pubescens* | dry leaves | Spain (Arboretum National des Barres, France) |
| *Buxus sempervirens* | dry leaves | France (B. Fady) |
| *Carpinus betulus* | dry leaves | France (B. Fady) |
| *Castanea sativa* | dry leaves | France (B. Fady) |
| *Cedrus libani* | dry leaves | Turkey (Common garden in France, B. Fady) |
| *Celtis australis* | dry leaves | France (B. Fady) |
| *Celtis tournefortii* | Herbarium (DNA) | Berlin Botanic Garden |
| *Ceratonia siliqua* | dry leaves | Italy (G. Bacchetta) |
| *Cercis siliquastrum* | dry leaves | France (B. Fady) |
| *Chamaerops humilis* | fresh leaves | France (B. Fady) |
| *Cotinus coggygria* | dry leaves | Montenegro (F. Médail) |
| *Corylus avellana* | dry leaves | France (B. Fady) |
| *Corylus colurna* | dry leaves | Turkey (Arboretum National des Barres, France) |
| *Cotoneaster granatensis* | dry leaves | Spain (J. Arroyo) |
| *Crataegus azarolus* | dry leaves | Cyprus (N. Eliades) |
| *Crataegus heldreichii* | dry leaves | Greece (MNHN, Paris Natural History Museum) |
| *Crataegus laciniata* | dry leaves | Algeria (MNHN, Paris Natural History Museum) |
| *Crataegus laevigata* | dry leaves | Arboretum National des Barres, France |
| *Crataegus monogyna* | dry leaves | France (B. Fady) |
| *Crataegus nevadensis* | Herbarium (DNA) | Berlin Botanic Garden |
| *Crataegus orientalis* | dry leaves | Armenia (MNHN, Paris Natural History Museum) |
| *Cupressus sempervirens* | dry leaves | France (B. Fady) |
| *Fagus orientalis* | dry leaves | Romania (D. Postolache) |
| *Fagus sylvatica* | dry leaves | France (B. Fady) |
| *Ficus carica* | dry leaves | France (B. Fady) |
| *Fraxinus angustifolia* | dry leaves | France (B. Fady) |
| *Fraxinus excelsior* | dry leaves | France (B. Fady) |
| *Fraxinus ornus* | dry leaves | France (B. Fady) |
| *Fraxinus pallisae* | herbarium | United Kingdom (MNHN, Paris Natural History Museum) |
| *Genista etnensis* | dry leaves | Italy (Arboretum National des Barres, France) |
| *Genista tyrrhena* | dry leaves | Italy (F. Médail) |
| *Ilex aquifolium* | dry leaves | France (B. Fady) |
| *Juglans regia* | dry leaves | France (B. Fady) |
| *Juniperus communis* | dry leaves | France (B. Fady) |
| *Juniperus drupacea* | dry leaves | Greece (Arboretum National des Barres, France) |
| *Juniperus foetidissima* | dry leaves | Cyprus (N. Eliades) |
| *Juniperus macrocarpa* | dry leaves | Spain (J. Arroyo) |
| *Juniperus navicularis* | dry leaves | Spain (J. Arroyo) |
| *Juniperus oxycedrus* | dry leaves | France (B. Fady) |
| *Juniperus phoenicea* | dry leaves | France (B. Fady) |
| *Juniperus thurifera* | dry leaves | Spain (J. Arroyo) |
| *Laurus nobilis* | dry leaves | France (B. Fady) |
| *Lonicera arborea* | dry leaves | Spain (Arboretum National des Barres, France) |
| *Malus florentina* | dry leaves | Italy (Arboretum National des Barres, France) |
| *Malus sylvestris* | dry fruit | Lebanon (F. Médail) |
| *Malus trilobata* | dry leaves | Spain (J. Arroyo) |
| *Myrica faya* | dry leaves | MNHN, Paris Natural History Museum |
| *Myrtus communis* | dry leaves | France (B. Fady) |
| *Nerium oleander* | dry leaves | France (B. Fady) |
| *Olea europaea* | dry leaves | France (B. Fady) |
| *Phillyrea angustifolia* | dry leaves | France (B. Fady) |
| *Phillyrea latifolia* | dry leaves | France (B. Fady) |
| *Phoenix theophrasti* | fresh leaves | Crete (B. Fady) |
| *Pinus brutia* | dry leaves | Turey (Common garden in France, B. Fady) |
| *Pinus halepensis* | dry leaves | France (B. Fady) |
| *Pinus heldreichii* | dry leaves | Serbia (B. Fady) |
| *Pinus mugo* | dry leaves | France (B. Fady) |
| *Pinus nigra* | dry leaves | France (B. Fady) |
| *Pinus pinaster* | dry leaves | France (B. Fady) |
| *Pinus pinea* | dry leaves | France (B. Fady) |
| *Pinus sylvestris* | dry leaves | France (B. Fady) |
| *Pinus uncinata* | dry leaves | France (B. Fady) |
| *Pistacia atlantica* | dry leaves | Cyprus (N. Eliades) |
| *Pistacia lentiscus* | dry leaves | France (B. Fady) |
| *Pistacia terebinthus* | dry leaves | Italy (G. Bacchetta) |
| *Populus nigra* | dry leaves | France (B. Fady) |
| *Prunus avium* | dry leaves | France (B. Fady) |
| *Prunus cocomilia* | dry leaves | Greece (Arboretum National des Barres, France) |
| *Prunus mahaleb* | dry leaves | France (B. Fady) |
| *Pyrus bourgaeana* | dry leaves | Spain (J. Arroyo) |
| *Pyrus cordata* | Herbarium (DNA) | Berlin Botanic Garden |
| *Pyrus elaeagrifolia* | Herbarium (DNA) | Berlin Botanic Garden |
| *Quercus alnifolia* | dry leaves | Cyprus (F. Médail) |
| *Quercus aucheri* | Herbarium (DNA) | Berlin Botanic Garden |
| *Quercus canariensis* | dry leaves | Spain (J. Arroyo) |
| *Quercus coccifera* | dry leaves | France (B. Fady) |
| *Quercus congesta* | dry leaves | Italy (G. Bacchetta) |
| *Quercus gussonei* | dry leaves | Italy (G. Bacchetta) |
| *Quercus ichnusae* | dry leaves | Italy (G. Bacchetta) |
| *Quercus ilex* | dry leaves | France (B. Fady) |
| *Quercus ithaburensis* | Herbarium (DNA) | Berlin Botanic Garden |
| *Quercus pubescens* | dry leaves | France (B. Fady) |
| *Quercus suber* | dry leaves | France (B. Fady) |
| *Rhamnus alaternus* | dry leaves | France (B. Fady) |
| *Rhamnus cathartica* | dry leaves | France (B. Fady) |
| *Rhamnus persicifolia* | dry leaves | Italy (G. Bacchetta) |
| *Rhododendron ponticum* | dry leaves | Spain (J. Arroyo) |
| *Salix amplexicaulis* | Herbarium (DNA) | Berlin Botanic Garden |
| *Salix apennina* | Herbarium (DNA) | Italy (F. Bagnoli) |
| *Salix arrigonii* | dry leaves | Italy (G. Bacchetta) |
| *Salix eleagnos* | dry leaves | France (B. Fady) |
| *Salix fragilis* | Herbarium (DNA) | Berlin Botanic Garden |
| *Salix gussonei* | dry leaves | Italy (G. Bacchetta) |
| *Salix salviifolia* | dry leaves | Spain (J. Arroyo) |
| *Salix xanthicola* | Herbarium (DNA) | Berlin Botanic Garden |
| *Searsia tripartita* | dry leaves | Algeria (F. Médail) |
| *Sorbus aria* | dry leaves | France (B. Fady) |
| *Sorbus austriaca* | dry leaves | Slovenia (T. Nikolić) |
| *Sorbus domestica* | dry leaves | France (B. Fady) |
| *Sorbus graeca* | Herbarium (DNA) | Berlin Botanic Garden |
| *Sorbus latifolia* | dry leaves | Italy (Arboretum National des Barres, France) |
| *Sorbus mougeotii* | dry leaves | France (Arboretum National des Barres, France) |
| *Sorbus umbellata* | Herbarium (DNA) | Berlin Botanic Garden |
| *Spartium junceum* | fresh leaves | France (B. Fady) |
| *Staphylea pinnata* | dry leaves | France (Arboretum National des Barres, France) |
| *Styrax officinalis* | dry leaves | France (Arboretum National des Barres, France) |
| *Tamarix africana* | dry leaves | Italy (G. Bacchetta) |
| *Tamarix arborea* | dry leaves | Italy (G. Bacchetta) |
| *Tamarix boveana* | dry leaves | Spain (J. Arroyo) |
| *Tamarix dalmatica* | dry leaves | Italy (G. Bacchetta) |
| *Tamarix laxa* | dry leaves | Macedonia (MNHN, Paris Natural History Museum) |
| *Tamarix mascatensis* | dry leaves | Spain (J. Arroyo) |
| *Tamarix parviflora* | dry leaves | Italy (G. Bacchetta) |
| *Tamarix ramosissima* | Herbarium (DNA) | Berlin Botanic Garden |
| *Tamarix smyrnensis* | dry leaves | Cyprus (N. Eliades) |
| *Tamarix tetragyna* | dry leaves | Italy (G. Bacchetta) |
| *Tamarix tetrandra* | dry leaves | Italy (G. Bacchetta) |
| *Taxus baccata* | dry leaves | France (B. Fady) |
| *Tetraclinis articulata* | fresh leaves | Spain (B. Fady) |
| *Tilia cordata* | dry leaves | Greece (T. Nikolić) |
| *Ulmus canescens* | fresh leaves | France (B. Fady) |
| *Ulmus glabra* | dry leaves | France (B. Fady) |
| *Ulmus laevis* | dry leaves | France (Arboretum National des Barres, France) |
| *Ulmus minor* | dry leaves | France (B. Fady) |
| *Ulmus procera* | dry leaves | France (B. Fady) |
| *Vitex agnus-castus* | dry leaves | Italy (G. Bacchetta) |
| *Zelkova abelicea* | dry leaves | Greece (MNHN, Paris Natural History Museum) |
| *Zelkova sicula* | dry leaves | Italy (G.G. Vendramin) |

***Supplementary Table 5: Summary of the nomenclature level at which each trait is estimated for each species when running the WOODIV_traits_table_generation script.*** *Details about the script can be found in the main text. “species”: the trait value for the species is the direct measure value is only one is available or the mean if several measures are available; “genus/family/order”: the trait value for the species is the mean of all the values available for all the species of the same genus/family/order; “-”: no value is estimated for the species with the current nomenclature level (higher nomenclature level can be included by changing the script code).*

| **spcode** | **Height** | **SeedMass** | **SLA** | **StemSpecDens** |
| --- | --- | --- | --- | --- |
| AALB | species | species | species | species |
| AAND | species | species | species | species |
| ABOR | species | species | genus | genus |
| ACAM | species | species | species | species |
| ACAP | species | species | genus | genus |
| ACEP | species | species | genus | genus |
| ACOR | species | species | genus | species |
| AGLU.agg | species | species | species | species |
| AHEL | species | species | genus | genus |
| AHIP | species | species | species | species |
| AHYR | species | species | genus | genus |
| AINC | species | species | species | species |
| ALUS | genus | genus | genus | genus |
| AMON | species | species | species | species |
| ANEB | species | genus | species | genus |
| AOBT | species | species | genus | species |
| AOPA | species | species | species | species |
| AORI | species | species | genus | species |
| APIN | species | species | species | species |
| APLA | species | species | species | species |
| APSE | species | species | species | species |
| AROH | genus | genus | genus | genus |
| ASEM | species | species | genus | genus |
| ATAT | species | species | genus | genus |
| AUNE | species | species | species | species |
| AVIR | species | species | genus | genus |
| BBAL | species | species | species | genus |
| BPEN | species | species | species | species |
| BPUB | species | species | species | species |
| BSEM | species | species | species | species |
| CAEO | species | family | family | family |
| CAOR | species | species | species | genus |
| CAUS | species | species | species | species |
| CAVE | species | species | species | species |
| CAZA | species | species | genus | species |
| CBET | species | species | species | species |
| CCOG | species | species | species | species |
| CCOL | species | species | genus | genus |
| CGRA | species | species | family | family |
| CHEL | species | genus | genus | genus |
| CHUM | species | species | species | - |
| CLAC | species | genus | genus | genus |
| CLAE | species | species | species | genus |
| CLIB | species | species | family | species |
| CMON | species | species | species | species |
| CNEV | species | genus | genus | genus |
| CPEN | species | species | genus | genus |
| CPLA | species | species | genus | genus |
| CPYC | species | genus | genus | genus |
| CROR | species | species | genus | genus |
| CSAT | species | species | species | species |
| CSEM | species | species | species | species |
| CSIA | species | species | species | species |
| CSIM | species | species | species | species |
| CTOU | species | genus | genus | species |
| EARB | species | species | species | species |
| FALN | species | species | species | species |
| FANG | species | species | species | species |
| FCAR | species | species | species | species |
| FEXC | species | species | species | species |
| FORI | species | species | genus | species |
| FORN | species | species | species | species |
| FPAL | species | species | genus | genus |
| FPHI | species | family | family | family |
| FSYL | species | species | species | species |
| GETN | species | species | family | family |
| GTYR | species | species | family | family |
| IAQU | species | species | species | species |
| JCOM | species | species | species | species |
| JDRU | species | genus | genus | genus |
| JEXC | species | species | genus | species |
| JFOE | species | species | genus | species |
| JMAC | species | species | genus | genus |
| JNAV | species | species | species | genus |
| JOXY.agg | species | species | species | species |
| JPHO | species | species | species | species |
| JREG | species | species | species | species |
| JTHU | species | species | genus | species |
| LARB | species | species | order | order |
| LNOB | species | species | species | species |
| LORI | species | species | - | - |
| MCOM | species | species | species | species |
| MDAS | genus | genus | genus | genus |
| MFAY | species | order | order | species |
| MFLO | species | genus | genus | genus |
| MSYL | species | species | species | species |
| MTRI | species | genus | genus | genus |
| NOLE | species | species | species | species |
| OCAR | species | species | species | species |
| OEUR | species | species | species | species |
| PALB | species | species | species | species |
| PANG | species | species | species | species |
| PATL | species | species | genus | species |
| PAVI | species | species | species | species |
| PBOU | species | species | species | genus |
| PBRI | species | genus | genus | genus |
| PBRU | species | species | genus | species |
| PCER | species | species | species | species |
| PCOC | species | genus | genus | genus |
| PCOR | species | species | genus | genus |
| PELA | species | species | genus | genus |
| PHAL | species | species | species | species |
| PHEL | species | species | genus | genus |
| PINI | species | species | species | species |
| PLAT | species | species | species | species |
| PLEN | species | species | species | species |
| PLUS | species | species | genus | species |
| PMAH | species | species | species | species |
| PMUG.agg | species | species | species | species |
| PONI | species | species | species | species |
| PORI | species | species | - | species |
| PPAD | species | species | species | species |
| PPIA | species | species | species | species |
| PPIR | species | species | species | species |
| PPYR | species | species | genus | genus |
| PSPI | species | species | genus | genus |
| PSYL | species | species | species | species |
| PSYR | species | species | genus | species |
| PTER | species | species | species | species |
| PTHE | species | species | family | - |
| PTRE | species | species | species | species |
| PWEB | species | species | genus | genus |
| QALN | species | species | genus | species |
| QAUC | species | genus | genus | genus |
| QCAN | species | species | species | genus |
| QCER | species | species | species | species |
| QCOC | species | species | species | species |
| QCON | species | genus | genus | genus |
| QCRE | species | genus | genus | genus |
| QDAL | species | genus | genus | genus |
| QFAG | species | species | species | species |
| QFRA | species | species | species | genus |
| QGUS | species | genus | genus | genus |
| QICH | species | genus | genus | genus |
| QILE | species | species | species | species |
| QINF | genus | genus | genus | genus |
| QITH | species | genus | genus | genus |
| QPET | species | species | species | species |
| QPUB | species | species | species | species |
| QPYR | species | species | species | species |
| QROB | species | species | species | species |
| QSUB | species | species | species | species |
| QTRO | species | species | species | genus |
| RALA | species | species | species | species |
| RCAT | species | species | species | species |
| RPER | species | species | genus | genus |
| RPON | species | species | species | family |
| SALB | species | species | species | species |
| SAMP | species | genus | genus | genus |
| SAPE | species | genus | genus | genus |
| SAPP | species | species | genus | genus |
| SARI | species | species | species | species |
| SARR | species | genus | genus | genus |
| SATR | species | species | genus | genus |
| SAUC | species | species | species | species |
| SAUS | species | genus | genus | genus |
| SCAP | species | species | species | species |
| SCIN | species | species | genus | species |
| SDOM | species | species | species | species |
| SELE | species | species | genus | genus |
| SETR | species | family | family | family |
| SFRA | species | species | species | species |
| SGRA | genus | species | genus | species |
| SGUS | species | genus | genus | genus |
| SJUN | species | species | family | species |
| SLAT | species | species | genus | genus |
| SMOU | species | species | genus | genus |
| SNIG | species | species | species | species |
| SOFF | species | species | order | species |
| SPED | species | genus | genus | genus |
| SPEN | species | species | species | genus |
| SPIN | species | species | - | - |
| SPUR | species | species | species | species |
| SRAC | species | species | genus | genus |
| SSAL | species | genus | genus | genus |
| STOR | species | species | species | species |
| SUMB | genus | species | genus | genus |
| SVIM | species | genus | species | genus |
| SXAN | species | genus | genus | genus |
| TAFR | species | species | genus | genus |
| TARB | genus | genus | genus | genus |
| TART | species | species | species | species |
| TBAC | species | species | species | species |
| TBOV | species | genus | genus | genus |
| TCAN | genus | genus | species | genus |
| TCOR | species | species | species | species |
| TDAL | species | genus | genus | species |
| TGAL | species | species | genus | genus |
| THAM | species | genus | genus | genus |
| TLAX | species | genus | genus | genus |
| TMAS | species | genus | genus | genus |
| TMIN | species | genus | genus | genus |
| TPAR | species | genus | genus | genus |
| TPAS | genus | genus | genus | genus |
| TPLA | species | species | species | species |
| TRAM | species | species | genus | genus |
| TSMY | species | genus | genus | species |
| TTEG | species | genus | genus | species |
| TTEN | species | species | genus | species |
| TTOM | species | species | genus | genus |
| UCAN | species | genus | genus | species |
| UGLA | species | species | species | species |
| ULAE | species | species | species | species |
| UMIN | species | species | species | species |
| UPRO | species | genus | genus | species |
| VAGN | species | species | species | species |
| ZABE | species | family | family | family |
| ZSIC | species | family | family | family |

***Supplementary figure 1****.* ***Geometric median tree of the 210 tree species of Euro-Mediterranean from Médail et al.***^1^  *based on the three plastid DNA regions matK, rbcL and trnH-psbA, computed from the set of 100 replicates pf phylogenetic trees built using the Simulation with Uncertainty for Phylogenetic Investigating (SUNPLIN) method*^73^*.*


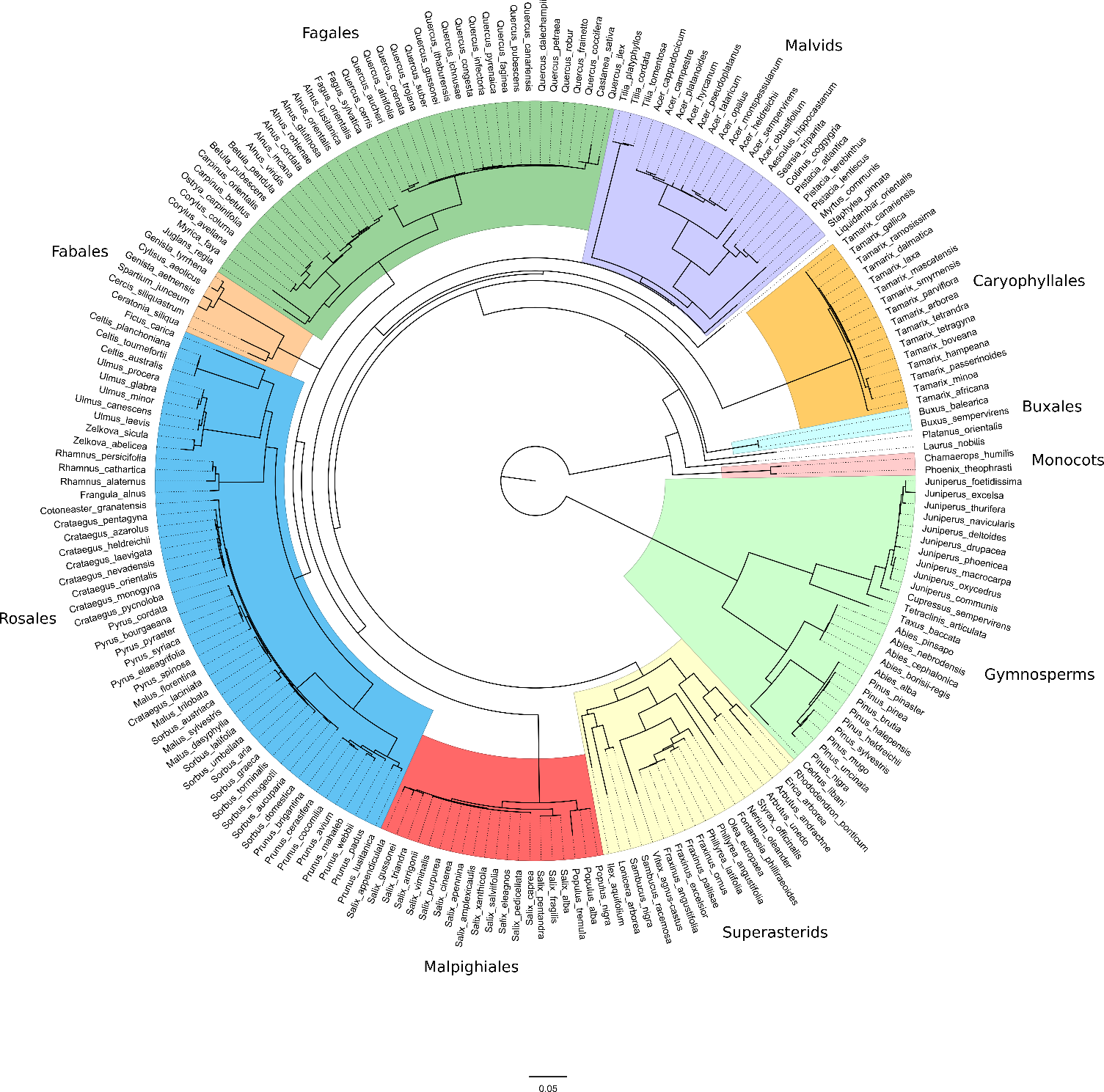

Supplement: Supplementary file 1 — Supplementary Information [file 41597_2021_873_MOESM1_ESM.docx]
